# Development of a novel air–liquid interface airway tissue equivalent model for in vitro respiratory modeling studies

**DOI:** 10.1038/s41598-023-36863-1

**Published:** 2023-06-22

**Authors:** Timothy Leach, Uma Gandhi, Kimberly D. Reeves, Kristina Stumpf, Kenichi Okuda, Frank C. Marini, Stephen J. Walker, Richard Boucher, Jeannie Chan, Laura A. Cox, Anthony Atala, Sean V. Murphy

**Affiliations:** 1 Wake Forest School of Medicine, Medical Center, Wake Forest Institute for Regenerative Medicine, 391 Technology Way, Winston-Salem, NC 27101 USA; 2 Wake Forest School of Medicine, Medical Center Boulevard, Virginia Tech-Wake Forest School of Biomedical Engineering and Sciences, Winston-Salem, NC 27157 USA; 3 Center for Precision Medicine, Department of Internal Medicine, Wake Forest School of Medicine, Winston-Salem, NC 27157 USA; 4 Marsico Lung Institute/Cystic Fibrosis Research Center, University of North Carolina at Chapel Hill, Chapel Hill, NC 27599 USA

**Keywords:** Tissue engineering, Biomaterials

## Abstract

The human airways are complex structures with important interactions between cells, extracellular matrix (ECM) proteins and the biomechanical microenvironment. A robust, well-differentiated in vitro culture system that accurately models these interactions would provide a useful tool for studying normal and pathological airway biology. Here, we report the development and characterization of a physiologically relevant air–liquid interface (ALI) 3D airway ‘organ tissue equivalent’ (OTE) model with three novel features: native pulmonary fibroblasts, solubilized lung ECM, and hydrogel substrate with tunable stiffness and porosity. We demonstrate the versatility of the OTE model by evaluating the impact of these features on human bronchial epithelial (HBE) cell phenotype. Variations of this model were analyzed during 28 days of ALI culture by evaluating epithelial confluence, trans-epithelial electrical resistance, and epithelial phenotype via multispectral immuno-histochemistry and next-generation sequencing. Cultures that included both solubilized lung ECM and native pulmonary fibroblasts within the hydrogel substrate formed well-differentiated ALI cultures that maintained a barrier function and expressed mature epithelial markers relating to goblet, club, and ciliated cells. Modulation of hydrogel stiffness did not negatively impact HBE differentiation and could be a valuable variable to alter epithelial phenotype. This study highlights the feasibility and versatility of a 3D airway OTE model to model the multiple components of the human airway 3D microenvironment.

## Introduction

The human bronchial tree is a complicated heterogeneous system that has important functions beyond being a simple barrier and conduit for air exchange. The airways are at the interface of the internal and external environment of the human body facilitating a variety of functions including mucociliary clearance, airway humidification, pathogen/particulate sensing and defense, and signaling to the underlying mesenchyme and immune system^1,2^. The airway epithelium is heterogeneous with distinct specialized cells including ciliated cells, goblet cells, club cells, basal cells, ionocytes, and neuroendocrine cells^2^. For many diseases there is dysregulation in these epithelial cell subtypes that directly corresponds with the disease including asthma, chronic obstructive pulmonary disorder, and cystic fibrosis^3,4^. Additionally, there is significant interplay with the pseudostratified epithelium and its basal three-dimensional (3D) microenvironment that includes subepithelial fibroblasts, immune cells, endothelial cells, and smooth muscle cells depending on airway size. Each of these cells found in the interstitial layer of the airways play key roles in the cell–cell communication that influence normal function and disease^5–7^. Besides the heterogenic cell populations within the airways, the subepithelial extracellular matrix (ECM) has been shown to play a key role in airway structural integrity as well as in cell regulatory functions such as cell activation, proliferation and differentiation^8^. These proteins and polymers are tissue-specific and provide specific ECM-cell receptor signaling. For instance, the composition of collagen within the airways along with the corresponding stiffness has been directly correlated to disease phenotypes and pathologically activated cells^9^. Despite advances in pulmonary medicine, we currently lack an optimal non-clinical model comprised of these important cell–cell, ECM-cell, and biomechanical components of the human airways.

The current gold standard for the study of the human airways is a monoculture of human bronchial epithelial (HBE) cells cultured on porous polymer membranes at an air–liquid interface (ALI)^1,10^. The main advantage of this model is the air interface which promotes HBE differentiation and pseudostratification. The ALI allows for mucus production and ciliation of the HBE as well as many other important functions such as barrier properties, aerosolized exposure, wound healing and regeneration. In conjunction with in vivo animal models, this model has provided many years of successful application to better understand normal airway epithelial function, disease, injury, and evaluation of therapeutics^11–13^. Still, this 2D ALI model has limitations, including: (1) lack of physiological cell–cell interactions with non-epithelial cells, such as the underlying stromal cells, (2) lack of cell-ECM matrix interactions beyond collagen coating of the polymer surface to mimic the basal membrane, and (3) a growth surface that is orders of magnitude stiffer than the native in vivo tissue microenvironment. These non-physiological conditions are likely to impact HBE phenotype, heterogeneity, and functionality in vitro resulting in an imprecise representation of human airway epithelial phenotype, altered pharmacodynamics, and limitations with modeling complex disease^1,14–20^. The absence of a 3D environment limits the ability to study diseased bronchial conditions due to the complex interactions of most conditions that involve the surrounding tissue environment^21,22^.

Therefore, there has been significant interest in the development of 3D cell culture models and microfluidic models to address these limitations and better represent the microenvironment experienced by cells in vivo. There have been two main approaches to date: spheroidal models and 2D planar microfluidic models. Spheroidal models address some limitations of 2D monoculture models by providing improved cell–cell interactions along with the possibility of an ECM-based microenvironment. A major drawback of the spheroidal design though is the lack of an external air liquid interface for analysis of the cilia and mucus along with aerosolized drug and toxin exposure. 2D planar microfluidic models have been developed to allow for physiological air and liquid flow on the apical and basal sides of the HBEs, respectively^23–25^. Some of these 2D planar microfluidic models include co-cultures of HBEs with other cell types, often plated on the underside of the polymer membrane or in parallel membranes within the same microfluidic chip^23,25,26^. A major limitation of this design is the lack of a biomechanical ECM environment beyond collagen coating of the stiff polymer membrane surface to support cell attachment. Therefore, there remains a critical need in the field for a 3D in vitro airway model that allows for both the formation of well-differentiated HBE cultures at ALI and a 3D microenvironment that includes physiological cell–cell, ECM-cell and biomechanical interactions.

**Figure 1:**
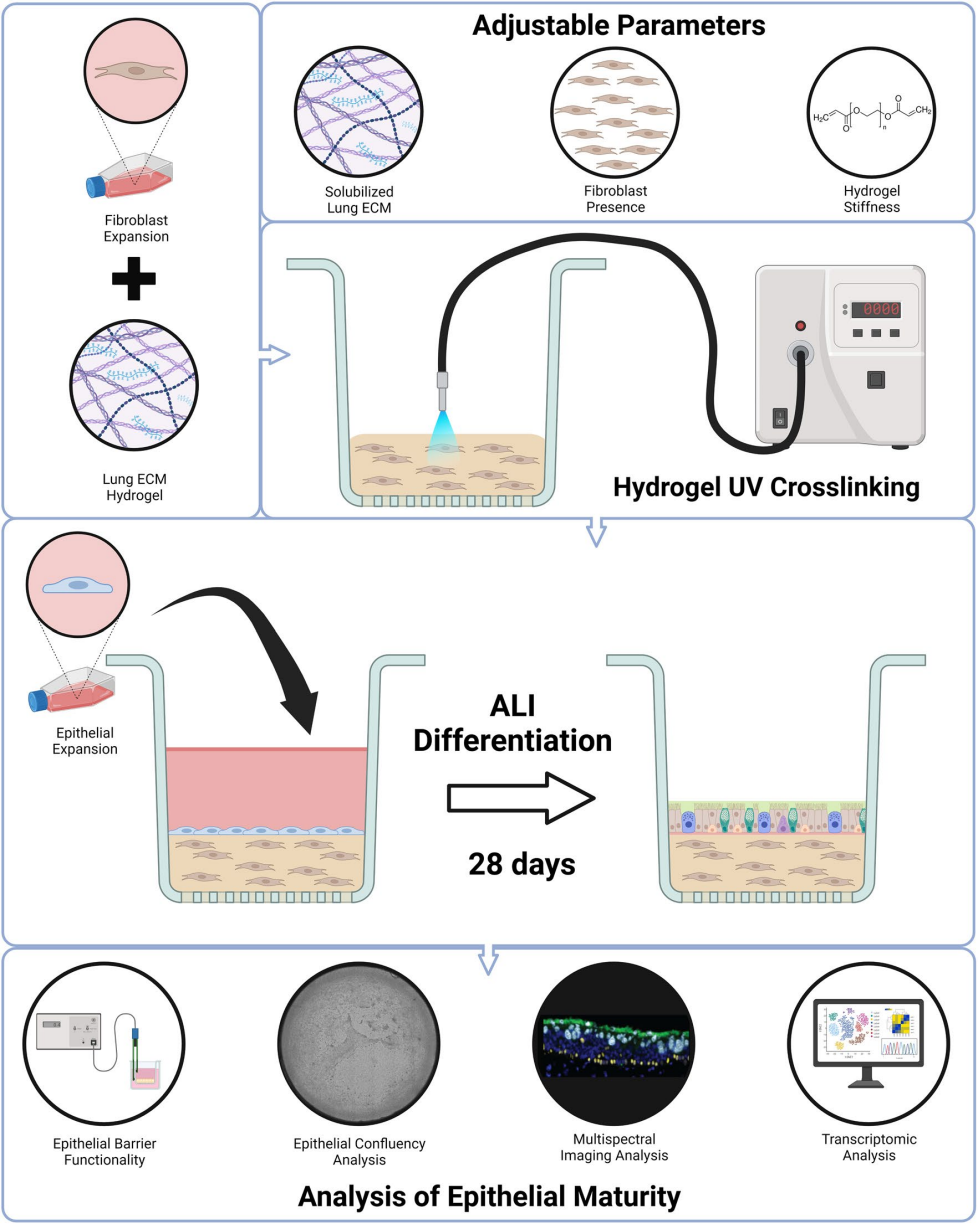
Organ tissue equivalent fabrication schematic. To fabricate the complete 3D OTE model, native lung fibroblasts were mixed with the hydrogel components and solubilized lung ECM and 80 μL was pipetted into an 8 μm polycarbonate membrane Transwell. After UV crosslinking, the HBEs were cultured on the apical side and allowed to differentiate at ALI for 28 days. The three main components of interest for the final OTE model are the native lung fibroblasts, solubilized lung ECM, and biomimetic hydrogel with stiffness adjusted via different crosslinkers. This figure was created using Biorender.com.

Here we report the development of a planar airway 3D organ tissue equivalent (OTE) model, comprised of a well-differentiated HBE layer at ALI, maintained on a hydrogel substrate layer that can contain native lung fibroblasts and solubilized human lung ECM (sECM) (Fig. 1). The interstitial hydrogel layer is composed of thiolated hyaluronic acid (HA), thiolated gelatin, and a polyethylene glycol (PEG) crosslinker that are photocrosslinkable for improved control of the biogel’s biomechanical properties. This system can be fabricated on standard transwells of any size or membrane pore size, or incorporated into microfluidic platforms^27,28^. We evaluated the incorporation of decellularized sECM and native pulmonary fibroblasts into the hydrogel layer, and compared a range of hydrogel stiffnesses representing physiological values expected in airway tissue. By varying the PEG crosslinker composition we can alter the elastic modulus of the hydrogels within a physiologically representative range of airway stiffnesses for healthy and pathological bronchial tissue without affecting the ECM composition of the hydrogel^29,30^. Meanwhile, the incorporation of human lung sECM and native lung fibroblasts are likely to provide airway-specific cytokine and ECM signaling molecules that improve HBE differentiation and attachment^31,32^. These model variants were characterized by measuring HBE growth, functionality, and differentiation compared to 2D ALI cultures and native human airway tissue samples.

## Results

### Solubilized lung extracellular matrix characterization

**Figure 2:**
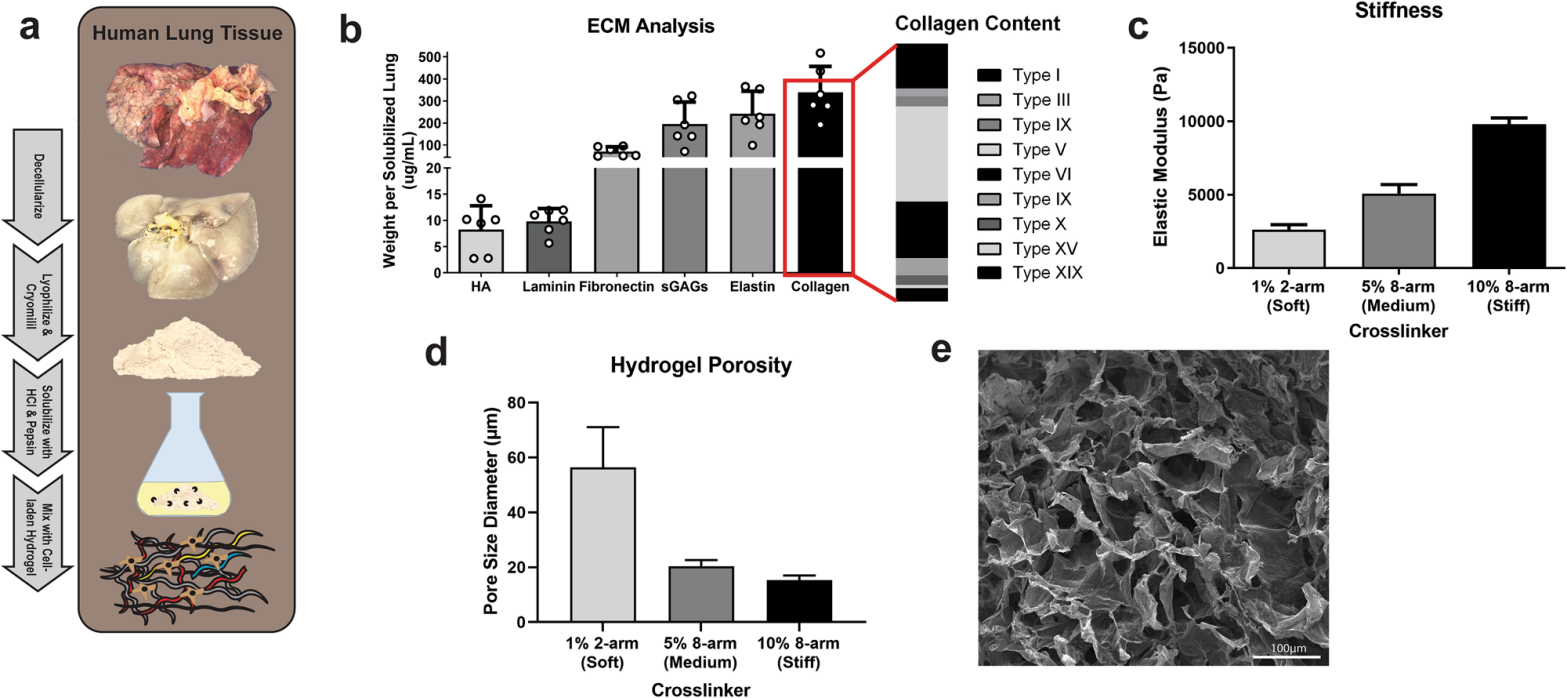
Fabrication & analysis of lung extracellular matrix-derived biogel. (**a**) To obtain the lung ECM biogel, human lung tissue is decellularized, lyophilized and cryomilled, digested, and mixed with a HA and gelatin-based hydrogel to form the biogel. (**b**) Utilizing ECM colorimetric kits and ELISAs, specific ECM components were quantified for each lung sample (n = 6). The ECM biogel can employ crosslinking molecules of different sizes and geometries to tune the biomechanical properties of the hydrogel, including elastic modulus (**c**) and pore size (**d**), measured via SEM imaging (**e**).

Organ ECM composition has shown to be tissue-specific^31^, and the composition could provide insight into crucial ECM components for well-differentiated HBEs that interact via cell adhesion and cell-ECM receptor signaling. We integrated decellularized lung sECM into the hydrogel component of our 3D OTE model, and evaluated its impact on HBE viability and a differentiated phenotype. Since the base hydrogel has been functionalized with heparin, it has the capability to bind a variety of proteins immobilizing them into OTE system including growth factors, laminin, tropoelastin, collagen, fibronectin, and vitronectin^33–37^. For instance, this specific hydrogel format successfully showed long-term release of vascular endothelial growth factor and basic fibroblast growth factor over several weeks^38^. A total of six healthy human lungs were decellularized and processed for analysis to provide a general characterization of the lung ECM microarchitecture (Fig. 2a). Complete decellularization was confirmed with no discernible nuclei or other cellular components after H&E staining (Suppl. Fig. S1). The sECM solution was quantified with assays for total protein and a variety of major ECM components to evaluate retention of these important components. After solubilization, between 3.3 and 5.4 mg/mL of total protein was recovered. We also quantified individual ECM components: collagen (338.8 ± 116.5 μg/mL), elastin (241.7 ± 111.9 μg/mL), laminin (9.80 ± 2.48 μg/mL), fibronectin (70.4 ± 21.1 μg/mL), sulfated glycosaminoglycans (sGAGs) (195.5 ± 103.4 μg/mL) and the major non-sulfated GAG, HA (8.2 ± 4.3 μg/mL) (Fig. 2b). Of the total collagen recovered, the majority of the collagen analyzed, based on spectral counts, was from the following types: type I (17.2 ± 3.2%), type V (36.4 ± 6.7%), and Type VI (21.7 ± 5.6%). After decellularization and solubilization, our human lung sECM retained multiple important ECM components known to be important for HBE growth, attachment and differentiation^39^. The highest concentration of lung sECM (~ 2 mg/mL of protein) that would allow for dissolution of the individual hydrogel components was utilized for fabricating hydrogels for all subsequent experiments in order to best mimic the ECM environment of the airways. Analysis of growth factors within the sECM was characterized, demonstrating that growth factors were retained and detectable in the sECM used in this study (Suppl. Table S1). The top 20 growth factors by concentration above the detectable limit and present in at least two sECM are shown.

### Hydrogel biomechanics characterization

The biomechanical properties of individual tissues have a significant influence on healthy and pathological cell populations and phenotypes. Substrate stiffness directly correlates with attachment of adherent cells and influences cell phenotype^40^. For airway tissue, measurements of airway biomechanical properties report a stiffness range of 1–20 kPa depending on bronchi generation and disease status^29,30,41^. For our study, hydrogels were fabricated to model healthy bronchi airway tissue stiffnesses with three hydrogel stiffness groups representing conditions we defined here as: Soft (2.6 kPa), Medium (5.1 kPa), and Stiff (9.8 kPa) (Fig. 2c). The stiffness of the hydrogel component was measured and confirmed via rheology using previously established protocols^31,42^. With an increase in stiffness, there was a corresponding decrease in porosity of the hydrogel as measured by scanning electron microscopy (SEM) imaging and quantification with average pore sizes of: 56.45 ± 14.65 μm (Soft), 20.47 ± 2.19 μm (Medium), and 15.20 ± 1.82 μm (Stiff) (Fig. 2d,e). There was no significant difference on the stiffness of the hydrogel if sECM was included in the hydrogel mixture (Suppl. Fig. S2). Increasing elastic modulus also correlated with increased storage modulus between the three stiffness groups (Suppl. Fig. S3). The ability to directly influence the hydrogel biomechanical properties without altering ECM concentrations provides a valuable tool for assessing their influence on HBE phenotype when cultured at ALI.

### Influence of 3D OTE parameters on HBE layer

To assess the impact of the individual components incorporated into the OTE model on HBE differentiation and functionality, we fabricated OTEs with hydrogels without either fibroblasts (FB) or lung sECM, with each individually incorporated into the hydrogel, and with both fibroblasts and lung sECM. The OTEs were fabricated with a density of 250,000 fibroblasts/OTE and a lung sECM protein concentration of 2 mg/mL to evaluate the feasibility of the OTE model. A seeding density of 1:1 for fibroblasts to HBE was used for our 3D OTE culture model based on epithelial to stromal cell counts from single cell analysis from literature^43^. These conditions were replicated across all three stiffness groups, as summarized below in Table 1. HBEs were cultured on the surface of each of these hydrogel groups, airlifted after 4 days, and assessed for differentiation at ALI 28. Each of these OTE groups were evaluated and compared to standard 2D ALI cultures and native human airway for all relevant and compatible analyses. Future studies will expand upon this design by evaluating concentration ranges for FB and lung sECM.

**Table 1:** Experimental groups.

Experimental groups (n = 24)				
	FB + /ECM +	FB +	ECM +	Neither
Soft (~ 2 kPa)	Group 1	Group 2	Group 3	Group 4
Medium (~ 5 kPa)	Group 5	Group 6	Group 7	Group 8
Stiff (~ 10 kPa)	Group 9	Group 10	Group 11	Group 12
2D culture	Group 13			

### Bronchial epithelial integrity and barrier function

**Figure 3:**
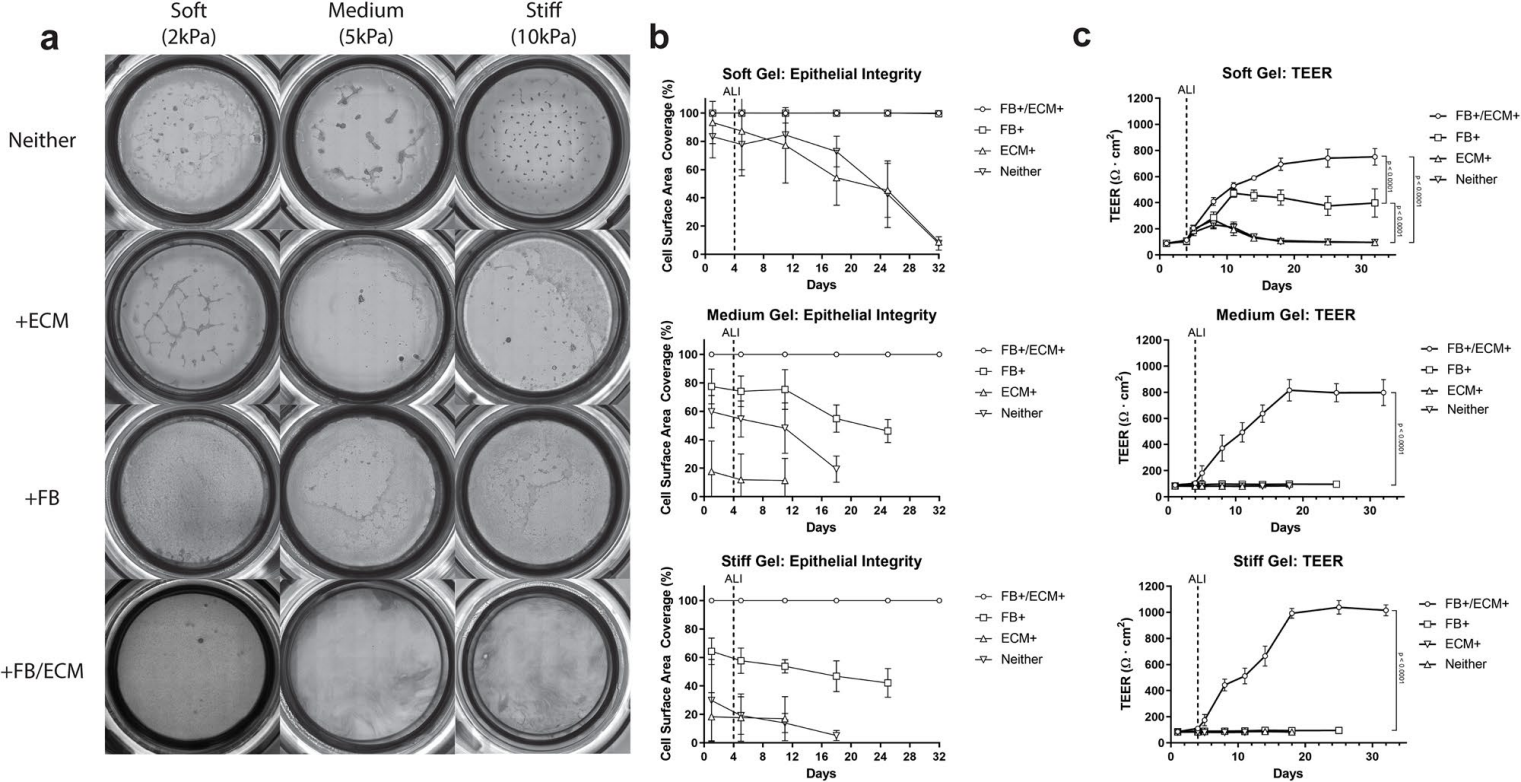
Epithelial barrier functionality analysis. (**a**) Representative whole mount images of the epithelial surface of the OTE cultures during the culture period. (**b**) Epithelial confluency was quantified and plotted for the duration of the culture (n = 6). (**c**) Trans-epithelial electrical resistance (TEER) of 3D OTE HBE cells during the entire culture period (n = 12). Significance compared the final day of TEER measurements and the vertical dotted line signifies the day the cultures were changed to ALI. (Note: Some error bars are too small to be shown).

A critical characteristic of ALI cultures is the epithelial barrier that functions to maintain an air–liquid interface and epithelial functionality. The standard assay to assess epithelial integrity and the corresponding polarization is measurement of the trans-epithelial electrical resistance (TEER). Epithelial integrity and barrier function was assessed by TEER and corresponding brightfield whole mount imaging to assess confluency of the epithelial monolayer (Fig. 3). We hypothesized that the presence of native FB and sECM would promote HBE attachment and subsequent development of tight junctions for an epithelial barrier.

Epithelial detachment and holes in the HBE monolayer could be observed in all OTE cultures that did not include both sECM and lung fibroblasts except for the FB + only group in the soft hydrogel group (Group 2) (Fig. 3a). The image quantification of the epithelium confluency illustrated > 99% confluency for these groups compared to all other groups which had < 50% confluency (Fig. 3b). Qualitatively, the cultures that were unable to maintain a confluent monolayer had HBE cells that either formed aggregates or spread out on the surface in contrast to their typical cobblestone phenotype (Suppl. Fig. S4). We expect that the inclusion of lung sECM along with the influence of native fibroblasts on the ECM microarchitecture, and potentially via production of supportive paracrine factors, provided a more favorable environment for cell attachment and basement membrane generation for a complete HBE monolayer.

TEER measurements supported the image quantification results with the cultures that did not include both FB and sECM dropping to near baseline resistance values at the end of their culture periods (Fig. 3c). In contrast, OTEs with both FB and sECM (Groups 1,5,9) had final TEER values more than 7X their baseline values indicating quality epithelial integrity and polarization compared to the other groups (p < 0.0001). The soft hydrogel with only FB (Group 2) did show a 4X increase in TEER from baseline but did not develop resistance values as large as the group with both FB and sECM (p < 0.0001). An important feature that was noted during the culture period of the OTEs was that none of the hydrogel models contracted from the edges resulting in the loss of the air–liquid interface and barrier function (Fig. 3a) typically seen with hydrogels with embedded fibroblasts.

TEER measurements were also taken for the 2D culture and similarly showed a 10X increase from its baseline value at the end of 28 days of ALI culture (Suppl. Fig. S5). When compared to the groups that did develop a quality monolayer barrier, the final TEER value of the 2D culture was significantly higher than all of the 3D OTE cultures (p < 0.001). This discrepancy is hypothesized to be due to the HBE interaction with the hydrogel and/or fibroblasts since hydrogel without epithelial cells did not change in TEER (Suppl. Fig. S6). Immunohistochemical fluorescent staining demonstrated the widespread presence of ZO-1 tight junction markers in both the 2D and 3D OTE cultures with both FB and ECM, confirming the barrier integrity shown by TEER measurements (Suppl. Fig. S7). Still, there is no in vivo standard of electrical resistance measurements for comparison to determine what an ideal physiological electrical resistance is for HBEs.

### Histological analysis of epithelial pseudostratification and differentiation

**Figure 4:**
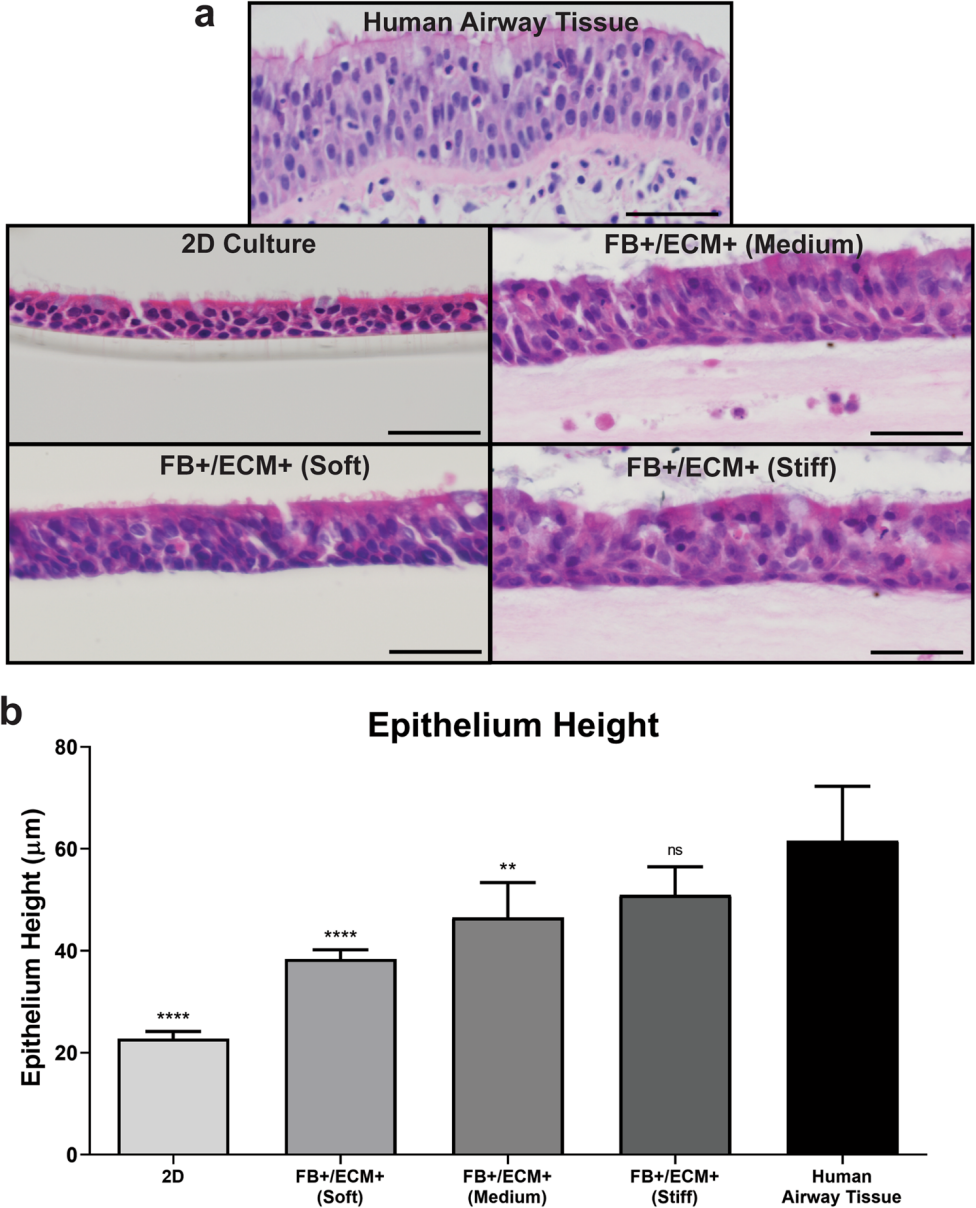
Epithelial pseudostratification analysis. (**a**) Representative H&E staining of the FB + /ECM + OTE model groups, 2D ALI Culture, and human airway tissue at ALI day 28. (Scale bar = 50 μm). (**b**) Quantitative analysis of the epithelial thickness at ALI day 28 completed via MATLAB image segmentation (n = 6). The significance is in relationship to human airway epithelium (*p < 0.05, **p < 0.01, ***p < 0.001, ****p < 0.0001).

Once OTE hydrogel conditions capable of forming confluent ALI cultures were established representing FB + /ECM + conditions of all three hydrogel stiffness variants, and FB + of the soft hydrogel variant (Groups 1, 2, 5 and 9), quantification of their capability to differentiate and HBE phenotype characterization was performed via histological, multispectral immunohistochemical, and RNA sequencing transcriptomic analysis. First, we performed H&E staining on the histologically processed cultures collected at the end of ALI culture and compared them to human airway tissue samples (Fig. 4a). Despite successful cultivation of a complete monolayer of HBE cells, not all groups evaluated demonstrated pseudostratification and qualitative signs of HBE differentiation. Unlike the groups that contained both FBs and sECM, the soft hydrogel with FB-only (Group 2) developed into a 2–3 multi-layered epithelium without clear signs of pseudostratification or ciliation as seen in human airway tissue (data not shown). On the other hand, the groups with both FB and sECM qualitatively showed pseudostratification. This was quantified by MATLAB analysis of the height of the HBE layer for 2D cultures, the OTE groups, and human airway tissue (Fig. 4b & Suppl. Fig. S8). When both FB and sECM were included in the OTE, there was a significant increase in epithelial height with regard to 2D ALI culture (Group1: 38.40 ± 1.79 μm, Group5: 47.31 ± 10.80 μm, Group9: 52.22 ± 11.72 μm, 2D: 22.78 ± 1.41 μm, p < 0.0001). Moreover, an increase in stiffness correlated with an increase in epithelial height. Specifically, when cultured on the Soft hydrogel (Group 1), the epithelial height was more closely matched with 2D ALI cultures and when cultured on the Stiff hydrogel (Group 9), the epithelial height was more closely matched with the human airway tissue (52.22 ± 11.72 μm vs 61.60 ± 10.70 μm, p < 0.01). While a larger range of stiffnesses needs to be evaluated before such conclusions can be made with confidence, a possible explanation for this change in epithelial height is the correlation of decreasing tissue stiffness with increasing bronchi generation^41^. This relationship provides an opportunity to develop and compare airway OTE models of different hydrogel stiffnesses for different epithelial phenotypes. Due to the lack of pseudostratification height and obvious signs of ciliated and goblet cells, only the FB + /sECM + OTE groups were used for multispectral and transcriptomic analysis of the differentiated HBE phenotype.

### Multispectral analysis of epithelial differentiation and functionality

**Figure 5:**
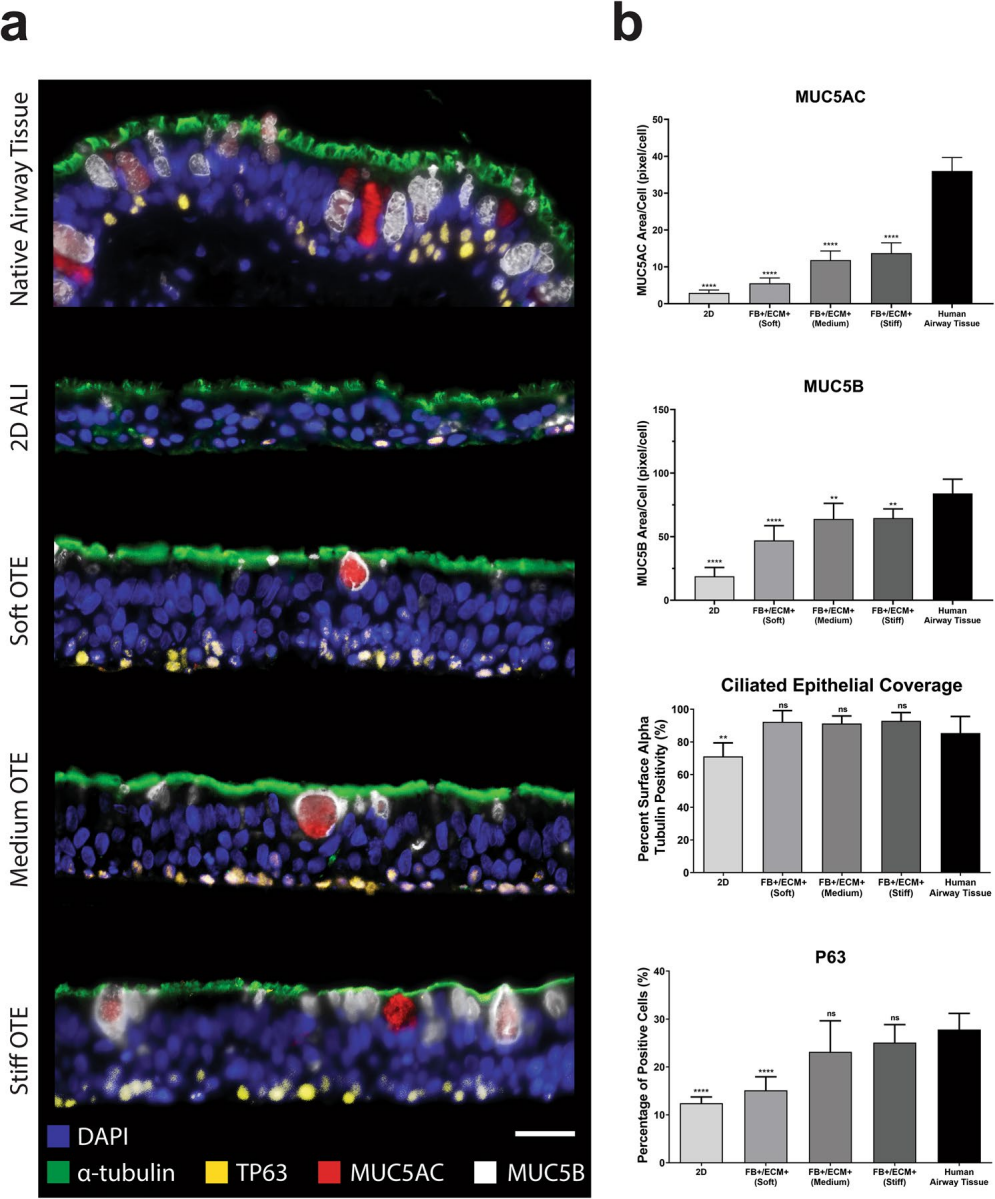
Multispectral analysis of epithelial differentiation markers. (**a**) Representative multispectral staining of human airway tissue, 2D ALI culture, and the complete OTE models for detection of the following markers: DAPI (blue), acetylated tubulin (green), MUC5B (white), MUC5AC (red), and TP63 (yellow) (Scale bar = 25 μm). (**b**) Associated quantitative analysis of HBE differentiation markers (acetylated tubulin, MUC5AC, MUC5B, and TP63) between the standard 2D ALI culture, OTE models, and human airway tissue (n = 6). (*ns* not significant, **p < 0.01, ***p < 0.001, ****p < 0.0001).

To characterize the heterogeneous population of differentiated cells in each OTE condition, a Nuance multispectral imaging system was used to quantify multiple molecular markers associated with the different HBE cell subtypes. This system allows for multiplexing and spectral unmixing of images to accurately quantify changes in molecular marker expression between samples while removing autofluorescent noise associated with the tissue, hydrogel, and native ECM. We quantified markers associated with the four major cell types of the airways: MUC5ac (goblet), MUC5b (club), alpha tubulin (ciliated), and TP63 (basal) (Fig. 5a). The remaining OTE groups, those that included both sECM and FB at varying stiffness (Groups 1, 5, and 9), and 2D ALI culture were compared against native human airway tissue. When qualitatively compared, all OTE cultures displayed cell type proportions comparable to human airway tissue, while the 2D cultures displayed decreased proportions of each of the evaluated cell types compared to all other groups (Fig. 5a). Quantitative analysis of these markers using the Nuance multispectral software demonstrated that all OTE cultures showed a significant increase in staining for each of the evaluated cell types compared to 2D ALI culture (p < 0.01) (Fig. 5b). OTE cultures had greater MUC5ac staining than 2D cultures (2D: 2.92 ± 0.77, 2 kPa: 5.52 ± 1.45, 5 kPa: 11.80 ± 2.49, 10 kPa: 13.69 ± 2.81), while all groups remained lower compared to human airway tissue (Airways: 25.99 ± 3.68). A similar trend was seen for the club cell marker MUC5b. OTE cultures had greater MUC5b staining than 2D ALI cultures (2D: 17.57 ± 6.58, 2 kPa: 47.08 ± 11.59, 5 kPa: 63.41 ± 13.30, 10 kPa: 63.55 ± 7.37, Airways: 84.04 ± 11.23). Ciliation of the HBE layer showed no statistical difference in ciliated epithelial coverage between human airway tissue and the OTEs, while 2D ALI samples had significantly lower ciliation than airway tissue and OTE groups (2D: 71.11 ± 8.30%, 2 kPa: 92.34 ± 6.83%, 5 kPa: 91.24 ± 4.65%, 10 kPa: 92.81 ± 5.20%, Airways: 85.39 ± 10.24%). Finally, the Medium and Stiff hydrogel groups were statistically similar in TP63 expression compared to airway tissue, while the Soft hydrogel group showed lower expression, statistically similar to 2D ALI cultures (2D: 12.43 ± 1.31%, 2 kPa: 15.15 ± 2.85%, 5 kPa: 23.15 ± 6.10%, 10 kPa: 25.07 ± 3.77%, Airways: 27.82 ± 3.38%). The quantified increase in each of the evaluated cell-type markers correlated with increasing OTE hydrogel stiffness. These results are indicative of the HBE layer on the OTE models representing a well-differentiated epithelium.

### Influence of 3D coculture microenvironment on differential epithelial transcriptomics

Transcriptomic profiles were developed to evaluate phenotypic differences between the three OTE models, 2D ALI culture, and previously published healthy human airway epithelial tissue^44^. Comparison of the top 10% expressed genes revealed a total of 556 genes shared between all culture models and the in vivo airway epithelium, which included most of the key genes of mature epithelium (Suppl. Fig. S9, Suppl. Table S2).

**Figure 6:**
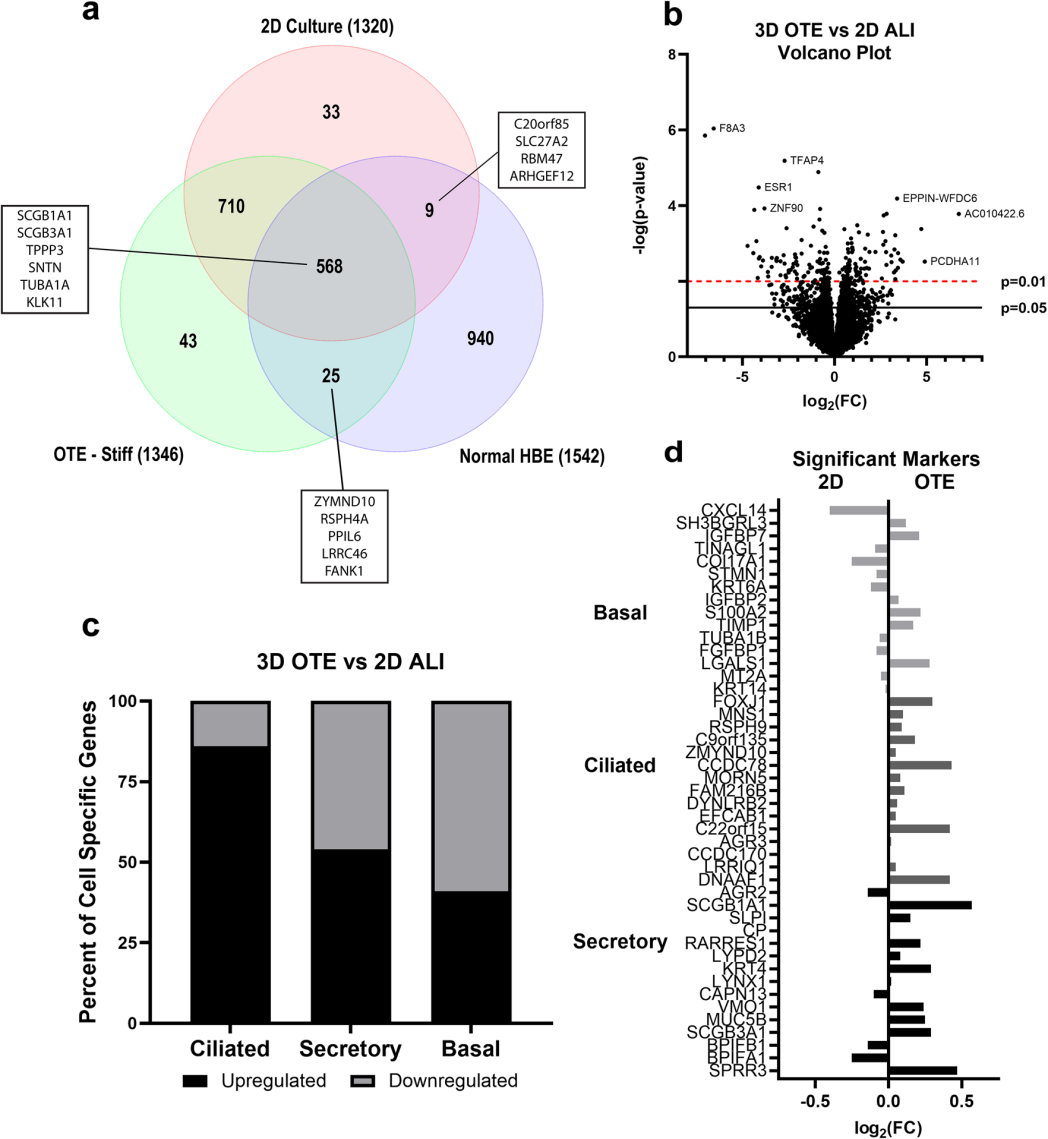
Well-differentiated HBE culture transcriptomics. (**a**) Venn diagram comparing the top 10% gene expression of the stiffest 3D OTE model, 2D ALI culture, and in vivo human airway epithelium (Normal HBE)^44^. (**b**) Volcano plot displaying differentially expressed transcripts of the stiff 3D OTE model in comparison to 2D ALI culture. (**c**) Expression regulation breakdown of the genes from the gene expression signatures of ciliated, secretory, and basal cells for the stiff 3D OTE model in comparison to 2D ALI culture. (**d**) Transcriptomic comparison of the fold change of the top 15 expressed gene markers related to HBE subtypes between the stiff 3D OTE model and 2D ALI culture (n = 3).

The stiffest OTE model was chosen for a more detailed comparison as it showed to be the most similar of the three OTE conditions to in vivo airway epithelium. When comparing the top 10% expressed genes, there was a total of 568 genes common to all three datasets, which included key genes of mature bronchial epithelium such as SCGB1A1, SCGB3A1, KLK11, TPPP3, TUBA1A, and SNTN (Fig. 6a, Suppl. Table S3). This demonstrates an important overlap in gene expression between in vivo airway epithelium, 2D ALI culture, and 3D OTE cultures. This is likely due to the considerable effect and advancement in cell culture media optimization and the ALI maturation methodology^45,46^. Interestingly, we identified 25 genes shared between the 3D OTE and human airway epithelium, which included several ciliogenesis markers related to mature epithelium such as ZMYND10, RSPH4A, and FANK1^47,48^. However, we also identified 710 genes shared by the 3D OTE model and 2D ALI culture, but not in vivo airway epithelium, indicating that both ex vivo cell culture methods may induce similar genetic phenotypes diverging from in vivo tissue.

To directly assess the cellular heterogeneity of the HBE between the 3D stiff OTE model and 2D ALI culture, we compared their transcriptomic profiles (Fig. 6b). We identified a total of 719 genes upregulated and 761 genes downregulated in the 3D OTE model compared to the 2D ALI culture (fold change > 1.5). From this dataset, the top expressed markers for the secretory, ciliated, and basal (cycling and non-cycling) cell phenotypes were identified from previous literature for phenotypic grouping^49^ (Suppl. Table S4). Goblet and club cell gene expression signatures were combined into a secretory phenotype group due to their similar gene profiles^49^. We found that 85% of the ciliated genes and 54% of the secretory genes were upregulated, while 59% of basal genes were downregulated in the 3D OTE models compared to 2D ALI culture (fold change > 1) (Fig. 6c). Expression levels of the top 15 genes of each of the gene sets are illustrated in Fig. 6d.

Gene pathway differences between the stiff 3D OTE model and 2D ALI model were analyzed using NetworkAnalyst. Significant differences (p < 0.05, fold change > 1.20) between the two groups were evaluated for signaling pathways (Kyoto Encyclopedia of Genes and Genomes [KEGG] and Reactome analysis) along with molecular functions and cellular components (PANTHER analysis)^50^. In total, there were 366 genes significantly upregulated and 333 significantly downregulated in the stiff 3D OTE model compared to the 2D ALI cultures. Using KEGG and Reactome pathway analysis, pathways of interest that were upregulated in the 3D OTE model included those relating to xenobiotic metabolism and tight junctions (Suppl. Table S5). Analysis with the PANTHER database demonstrated upregulation of genes associated with cilia and microtubule binding/motor activity indicating a possible upregulation in cilia function (Suppl. Table S5). Meanwhile, there were no specific pathways or components of interest that were downregulated in the 3D OTE model compared to 2D ALI culture.

## Discussion

In this study, we describe the development of a novel 3D OTE human airway model on a biomimetic hydrogel substrate, with incorporated native lung fibroblasts and solubilized lung ECM that can maintain ALI. We evaluated the influence of these biomimetic variables on important phenotypic characteristics that define a well-differentiated human airway epithelium. We found that the OTE model is capable of developing a well-differentiated polarized HBE layer epithelium and provides the versatility to adapt these variables to gain a better understanding of human airway tissue and cellular function in vitro.

The inclusion of native lung fibroblasts and solubilized lung ECM were essential components for HBE attachment, viability, pseudostratification and differentiation on the hydrogel substrate. Critical phenotypic and functional characteristics were observed in these complete OTE models: increased pseudostratification, increased TEER, and expression of markers for ciliated, goblet, club and basal cells. Transcriptomic analysis confirmed that the functional genomic signature of the 3D OTE HBE layer was characteristic of a mature, well-differentiated epithelium.

One of the main novel components of this OTE is the functionalized hydrogel interstitial layer comprised of thiol-modified HA and gelatin that can incorporate solubilized decellularized human lung ECM. The thiol-modified base structure of this hydrogel provides a covalently linked backbone to minimize hydrogel contraction. This backbone can also be modified to adjust its biomechanical properties without significantly affecting the total ECM content. Unlike many other hydrogel formats, this allows for independent examination of this aspect of the microenvironment. For these reasons functionalized hydrogels have been applied successfully in multiple other regenerative medicine applications^31,38,51–53^. We illustrated here that HBE can successfully form a monolayer and mature phenotype after 28 days under ALI conditions on hydrogels fabricated within a stiffness range of 2–10 kPA in our 3D multicellular OTE model. Histological analysis of the HBE layer demonstrated that the different stiffness hydrogel substrates affected the level of epithelial thickness. While more work is needed to evaluate this phenomenon, hydrogels matching the stiffness of the proximal or distal airways might be an important driver of HBE phenotype and heterogeneity observed across these tissue locations. Mechanical cues have been linked to the biological patterning and branching morphogenesis of the developing lung^54^. Additionally, while not investigated here, this hydrogel composition can reach stiffnesses as high as 20 kPa^31^, approximating the range of fibrotic airway stiffness^55^. Stiffer substrates have shown to promote myofibroblast activation and epithelial dysregulation seen in fibrotic phenotypes. For instance, altered mechanical properties are key characteristics of diseases like asthma and pulmonary fibrosis^56^. Future work will examine whether increasing the hydrogel stiffness to approximate fibrotic tissue stiffness results in fibrotic behaviors from the epithelium and/or fibroblasts including matrix deposition, epithelial-mesenchymal transition, and fibrotic transcriptomic changes.

The second crucial component of our OTE model is the incorporation of tissue-specific ECM to achieve a more physiological relevant biochemical environment. We have previously demonstrated successful isolation of ECM components from our decellularization process and their beneficial effects on the native cell types^31^. Our results indicate that the inclusion of lung sECM was crucial for the formation and maturation of the HBE monolayer on our functionalized hydrogel system. While HBE cells have been cultured on individual ECM substrates, such as collagen^57^ and fibrin^58^, they do not provide the same natural attachment points and biochemical cues that influence biological activity seen in tissue-specific ECM^59^. Moreover, current hydrogel formulations lack the discrete ECM quantities seen in our sECM, such as laminin which promotes epithelial attachment, modulate cell behavior, and stimulate basement membrane generation^60^. Although the mechanisms are not fully understood, our results indicate that airway-specific ECM not only promotes epithelial attachment, but also differentiation on an otherwise unsuitable hydrogel surface. Our future studies will evaluate fabrication of a “defined” biomimetic hydrogel including individually incorporated ECM components at ratios and concentrations identified in our analysis. This may help to mitigate the challenges and resources involved in sourcing and isolating human lung sECM, as well as donor variability regarding ECM concentrations. However, the evaluation of patient-, tissue location- or disease-specific sECM on OTE phenotype is also an area worth exploring. Subsequent analyses will also include characterizing the hydrogel base immobilization and release of crucial growth factors and ECM components within the sECM.

A third major component of our 3D OTE model is the inclusion of native lung fibroblasts within the interstitial hydrogel matrix. Similar to the addition of lung sECM, our results highlight the importance of native lung fibroblasts in the formation and maturation of the HBE monolayer on our functionalized hydrogel system. Previous studies have described a symbiotic relationship between the HBE layer and the underlying mesenchyme^61,62^. This beneficial effect on the HBEs is expected to be mainly based on paracrine signaling since there is minimal direct contact of the fibroblasts with the HBE layer throughout the hydrogel in our model, although we cannot rule this out as a contributor. Our results indicate that the combination of sECM with native lung fibroblasts provided the optimal microenvironment for HBE viability and maturation. This leads us to believe that there is an interaction between the sECM and fibroblasts, and the fibroblasts may reorganize the surrounding ECM into a more favorable environment for HBEs. Within a hydrogel environment, lung fibroblasts have shown to secrete ECM and promote improved attachment of HBEs^32^. Similar to the differential sourcing of lung sECM, we can use pathological sources of fibroblasts to showcase their interdependent relationship with the HBE. The inclusion of native lung fibroblasts in the OTE will be a valuable component for pathological phenotypes where fibroblasts play a major role such as pulmonary fibrosis and COPD^20^.

To evaluate differentiation of the HBE layer, the main functional cell types of the airway epithelium were quantified via multispectral analysis. For each cell type quantified, OTEs demonstrated higher expression when compared to 2D culture, regardless of hydrogel stiffness. For ciliation, MUC5b expression, and TP63 expression, our 3D OTE models demonstrated more similar staining levels to the human airway epithelium compared to 2D ALI cultures. MUC5ac was expressed at a significantly higher level compared to 2D ALI cultures, but still lower than levels observed in human airway epithelium. A possible reason for the lower expression of these characteristic markers in 2D ALI cultures is that they have shown to have a greater population of undifferentiated columnar epithelium compared to epithelia recovered from in vivo airway brushing^45^. Expression of these markers are important characteristics of well-differentiated bronchial epithelium and are functionally important for normal epithelial function. For example, ciliation expression and mucin production are crucial components of the airways defense mechanism to potentially harmful xenobiotics and gases^63^. Meanwhile, TP63 expression is a key characteristic of the multipotent capacity of the bronchial epithelial to undergo wound repair^64^.

We also quantified the transcriptomic changes between the 3D OTE and 2D ALI culture using RNA sequencing. Comparison of the transcriptomic profile of the 3D OTEs with healthy airway epithelial tissue illustrated that epithelium grown on the 3D OTEs is capable of differentiating into a mature epithelium. Not surprising, the 3D OTE cultures shared a large amount of transcriptomic similarity to 2D ALI culture and showed similar genotypic profiles with regard to the different epithelial subtypes. However, supporting findings from the multispectral IHC of functional cell types, 3D OTE transcripts showed slightly increased expression of genes relating to ciliated (FOXJ1 +) and club cells (SCGB1A1 + /MUC5B +). Interestingly, the gene set correlating to basal cells downregulated in the 3D OTE model compared to 2D ALI culture, in contradiction to the TP63 IHC quantification. This disparity could be explained by different subpopulations of basal and suprabasal cells in the airways and culture samples, or the fact that some club cells have shown to express TP63^49,65^. While TP63 is an identifying marker for progenitor basal cells, research has shown other basal cell subpopulations and suprabasal cells that are negative for TP63, but maintain many other markers, such as KRT16 and MKI67, which were upregulated in our 2D ALI cultures^66^.

The functional pathway analyses of genes differentially expressed between the 3D OTE model and standard 2D ALI culture identified several pathways of interest. Each of the databases provided results that supported greater xeniobiotic metabolism and cytochrome P450 activity in the 3D OTE model, which may indicate a greater population and activity of club cells^65^. This is supported by the upregulation in the SCGB1A1 gene expression and multispectral staining. Previously published analysis also found that 2D ALI culture show decreased expression of pathways related to xenobiotic metabolism when compared to in vivo airway tissue^45^. This study postulated that this difference could be explained by the consistent exposure to the external environment by in vivo epithelium, but our model indicates that a co-culture 3D ECM microenvironment may also play a role. We also identified upregulation of microtubule activity and cilia expression with PANTHER, supporting our other finds showing a greater degree of ciliation in the 3D OTE culture compared to 2D ALI culture. Interestingly, the pathway and cellular component analysis demonstrated increased tight junction expression in the 3D OTE model. Further investigations in the differences in tight junction formation may provide clarity in the differences in TEER measurements between the 3D OTE model and 2D ALI culture. A variety of factors including media composition, fibroblast co-culture, and the presence of a hydrogel have shown to alter the TEER with data referencing the corresponding differences in tight junction expression and electrogenic ion transport^67–69^. It is currently unclear what TEER values may be physiologically relevant due to challenges in measuring electrical resistance of in vivo or ex vivo human bronchial epithelial tissue for comparison.

While the permeable insert culture system is the current gold standard for in vitro airway research, one of the limitations of the current work is the lack of physiological basolateral liquid flow or apical airflow that could be achieved with a microfluidic platform. Our ongoing and future studies are focusing on incorporating this 3D OTE system into a microfluidic platform to assess these variables, as well as incorporating endothelial cells on the basolateral side for a more comprehensive model of the human airways. Translation towards a microfluidic platform for physiological flow will provide improved utility of the model for disease modeling and applications with aerosolized toxicants and drugs. In addition, we hope to gain an improved understanding of the impact of HBE culture variables we have established here, which includes generation of 3D OTEs using cells different donors, as well as direct from tissue isolation to evaluate the expected changes in cell phenotype and gene expression caused by initial tissue culture plastic establishment and expansion. Evaluation of sECM and fibroblast concentrations will be an important next step, as will be further dissecting the exact composition of ECM components most suitable for HBE function.

Our 3D multicellular human airway OTE allows for the evaluation of a variety of physiologically relevant parameters that can impact HBE differentiation and function. This provides a novel 3D airway model as a viable alternative or supplement to the gold standard of 2D ALI culture, as it allows for more complex and robust experimentation of human airway analogs in vitro. It is important to note that 2D ALI cultures still provide well-differentiated monocultures of bronchial epithelium that may suffice for many types of studies, and the model developed here can be used to supplement 2D ALI cultures. For instance, it could be anticipated that following an insult or disease, differences between the 3D OTE model and 2D ALI culture could highlight important cell–cell or cell-ECM interactions relevant for disease or injury mechanisms. We believe that the capability to alter and evaluate multiple physiological components, such as cell–cell, ECM-cell and substrate stiffness, provides a unique platform for further improvements of in vitro models of the human airway. The versatility of this model allows for the adjustment of typically inalterable variables in standard 2D monoculture for disease modeling such as: pathological ECM ratios related to diseases like asthma or idiopathic pulmonary fibrosis, fibroblast influence on normal and pathological epithelial behavior, ECM degradation and deposition, and environmental stiffness influence on epithelial phenotype. This 3D OTE model provides important and physiologically relevant variables for experimentation and opportunities to examine pathological phenotypes and immune responses not capable in current in vitro models.

## Materials and methods

### Lung extracellular matrix fabrication & analysis

Non-diseased, non-smoking human lung donor tissue was obtained (International Institute for the Advancement of Medicine), decellularized, and solubilized following protocols previously published by our group^31,33,70^. The solubilized lung ECM (sECM) was aliquoted and stored at -80 °C until further use. The ECM was diluted for colorimetric assays to measure collagen, elastin, sulphated glycosaminoglycan, and hyaluronan content (Biocolor, United Kingdom). Fibronectin and laminin were quantified using the Human Laminin ELISA kit (Abcam, United Kingdom) and Human Fibronectin ELISA Kit (R&D Systems, Minneapolis, MN), respectively. To estimate spectral counts of specific collagen types and the composition of growth factors, the sECM was sent to and processed by RayBiotech (Peachtree Corners, GA) using the Human L3 Glass Slide Array and Human Growth Factor Array Q1. Additionally, total protein content of the sECM was quantified with a Pierce™ BCA Protein Assay Kit (Thermofisher, Waltham, MA). A total of six individual lung donors were decellularized, solubilized, and analyzed for the solubilized ECM content.

### Fabrication of lung ECM hydrogel

The thiolated gelatin and hyaluronic acid components from HyStem-HP hydrogel kits (Heprasil and Gelin-S, Lot 18E023, ESI-BIO, Alameda, CA) along with a polyethylene glycol (PEG)-based crosslinker were dissolved in a 1:1 solution of sECM and a photoinitiator solution (P-ECM). The photoinitiator (4-(2-hydroxyethoxy)phenyl-(2-propyl)ketone, Sigma) was dissolved in deionized water to create a 0.1% w/v solution. Briefly, Heprasil and Gelin-S were dissolved in the P-ECM to create solutions of 2% w/v. The three components were mixed in a ratio of 2-parts Heprasil, 2-parts Gelin-S, and 1-part crosslinker. The following crosslinkers were tested for analysis: the linear PEG diacrylate (PEGDA) Extralink crosslinker from the HyStem kit at 1% w/v, and an 8-arm 10 kDa PEG Alkyne crosslinker (Creative PEGWorks, Chapel Hill, NC) at 5% and 10% w/v. After encapsulation of the cells, the final hydrogel solution was irradiated with ultraviolet light (365 nm, 18w/cm2, BlueWave 75 UV Light Curing Spot Lamp, Dymax, Torrington, CT) for crosslinking.

### Hydrogel material analysis

Cell-free hydrogels were prepared, with and without lung ECM, and analyzed for rheology and pore size. The hydrogels were mechanically tested at room temperature with a Discovery HR-2 Rheometer fitted with a steel 8 mm parallel plate geometry. The plate geometry was incrementally lowered into the hydrogel until a normal force of about 0.01N was achieved. To determine the elastic modulus, a compression test was run at a rate of 10 μm/s for a distance of 1.5 mm or until the hydrogel fractured, as previously described^42^. Data were collected every 25 ms for stress, force, and gap distance measurements to calculate the strain. The stress–strain measurements were utilized to establish a characteristic curve to calculate the elastic modulus. Additionally, a strain sweep test was run from 0.02 to 100% shear strain at an oscillation frequency of 1 Hz, during which the storage modulus was recorded, as previously described (n = 6)^31^.

For pore size, hydrogels were fabricated and allowed to swell in a phosphate buffered solution (PBS). After swelling, the samples were frozen and lyophilized overnight. The lyophilized gels were mounted onto an SEM stub, sputter-coated with 4 nm of gold-platinum, and imaged at 5 kV using a scanning electron microscope. The SEM images were analyzed with ImageJ (NIH) to quantify pore size (n = 3).

### Cell culture

Native human lung fibroblasts (Lonza, Basel, Switzerland) were grown in Gibco™ Minimal Essential Media Alpha (Thermofisher, MA, USA) supplemented with 10% fetal bovine serum (FBS), 1% Pen-Strep, and 1% L-glutamine. Primary HBEs (Marsico Lung Institute at the University of North Carolina, Chapel Hill) were cultured in non-proprietary UNC bronchial epithelial growth media (BEGM) on collagen type I-coated dishes. An irradiated fibroblast feeder cell layer and ROCK inhibitor (Y-27632) was supplemented to the culture as previously described^71,72^. At passage 4, HBEs were utilized for 2D ALI culture and OTE culture. At 70–90% confluency the cells were double trypsinized, counted and seeded on Millicell inserts and OTE cultures. A single donor for both the primary HBEs (DD057o) and native human lung fibroblasts (0000543644) were used for all experiments to establish the 3D OTE design.

### 2D ALI culture

HBEs were seeded on 0.4 μm pore size Millicell inserts (Millipore Sigma, PIHP01250, Billerica, MA) coated with collagen-I at a density of 4.15 × 10^5^ cells/cm^2^. The cells were cultured in non-proprietary “UNC ALI” media (Marsico Lung Institute at the University of North Carolina, Chapel Hill), 300 μL in the apical compartment and 3 mL in the basal compartment. At 100% confluency, the cultures were switched to ALI culture by aspirating the apical media and providing only basal media to the culture. Confluency of the cultures were confirmed with trans-epithelial electrical resistance (TEER) using STX2 chopstick electrodes connected to an EVOM2 voltmeter (World Precision Instruments, FL, USA). The apical surface of the culture was washed with PBS once a week to clear mucus.

### 3D organ tissue equivalent (OTE) fabrication

The OTE model was fabricated on 8 μm pore size Millicell inserts (Millipore Sigma, PI8P01250, Billerica, MA). Briefly, 80 μL of the lung ECM hydrogel containing 250,000 lung fibroblasts was pipetted into the apical side of the insert and irradiated with UV light. Two days after UV crosslinking of the hydrogel, HBEs were seeded on the apical side of the hydrogel at a density of 4.15 × 10^5^/cm^2^. The OTEs were maintained in the exact manner as 2D cultures and confluency of culture was confirmed with imaging and TEER. The apical surface of the culture was washed with PBS once a week to clear mucus, if necessary.

### Epithelial surface area coverage

To quantify the attachment and coverage of the HBEs between the groups, the HBE cell layer was imaged in brightfield utilizing an inverted Olympus IX83 microscope. The entire surface of the OTE culture was scanned at 10X and stitched together using CellSens software. The percent epithelial coverage of the surface of the culture was quantified from the stitched images (n = 5).

### Trans-epithelial electrical resistance (TEER)

TEER was measured using STX2 chopstick electrodes connected to an EVOM2 voltmeter. For resistance measurements, 300 μL of media was temporarily added to the apical side of the inserts. Data is presented as mean + / − standard deviation resistance values (n = 12) standardized to insert size (Ω·cm^2^). Statistical analysis was completed to compare the resistance values of the final day of culture. There was no statistical difference in resistance measurements between a blank 0.4 μm Millicell insert used for 2D ALI culture (90.2 ± 1.5 Ω·cm^2^) and a blank 8 μm Millicell insert used for 3D OTE culture (92.7 ± 0.9 Ω·cm^2^). To obtain the final resistance values, the blank value was subtracted from the total resistance of the sample.

### Histology and multispectral immunofluorescence

After 28 days at ALI, 2D and 3D ALI cultures were washed with PBS, fixed in 4% paraformaldehyde, embedded in paraffin, and 4 μm sections were prepared as previously described (n = 6)^15^. Additionally, sections from donated lung samples (n = 3) were fixed, embedded, and sectioned similarly for staining. Hematoxylin and eosin (H&E) and immunostaining were performed on the sections. MATLAB code was utilized to segment the H&E images to quantify epithelial height.

For immunostaining, the following antibodies were utilized: anti-MUC5AC antibody (1:250; ab212636; Abcam), anti-MUC5B antibody (1:2000; University of North Carolina, Chapel Hill), anti-TP63 antibody (1:300; ab76013; Abcam), anti-acetylated α-tubulin antibody (1:5000; T7451; Sigma). Multispectral IHC staining was performed using the serial staining Opal™ 7-color Manual IHC Kit with the following fluorescent markers: Opal 520, Opal 570, Opal 620, and Opal 690. Images were captured and processed using the Nuance Multispectral Imaging System and CellSens Software on Olympus BX-63 upright microscopes. Individual stains of each marker and their associated fluorophore were imaged and utilized to establish a spectral library. Spectral unmixing of the multiplexed panel was performed by Nuance software, and quantitation of the differentiation markers was calculated for the epithelium. Imaging and quantification excluded the edges of cultures to avoid any edge effect seen from the meniscus in the cultures.

### RNA isolation, library preparation, sequencing, and analysis

For RNA extraction and isolation, the surface of the HBE cells was washed with PBS two times prior to RNA collection to remove mucus. The cells were collected, centrifuged, and treated using the Direct-zol RNA Miniprep Plus Kit (Zymo Research, Irvine, CA) to isolate the RNA according to the manufacturer’s instructions (n = 3). RNA was quantified using the DeNovix RNA Assay Kit (DeNovix®, Wilmington, DE), and the RNA integrity was determined with an RNA ScreenTape Kit on the TapeStation 2200 system (Agilent, Santa Clara, CA). cDNA libraries were prepared from RNA extracts with a minimum RNA integrity number (RIN) of 9.5 using the NEXTFLEX® Combo-Seq mRNA/miRNA Kit (PerkinElmer®, Waltham, MA). Libraries were quantified using a KAPA Library Quantification Kit (Roche Sequencing and Life Science, Indianapolis, IN) with average fragment length determined by a DNA ScreenTape Kit on the TapeStation 2200 system (Agilent). Finally, cDNA libraries were normalized prior to pooling, and the pooled libraries were sequenced using an Illumina® NovaSeq 6000 system (Illumina®, Inc., San Diego, CA), generating an average of 30 M 1 × 101 bp reads per sample. Raw sequence reads were imported into Partek® Flow® software (Partek®, St. Louis, MO) for analysis. Cutadapt was used to trim the random barcode (4 bases) from the 5’ end and adapter (AAAAAAAAAA) from the 3’ end of the reads^73^. Bases with a Phred quality score < 20 were trimmed, and reads < 15 bases in length were discarded. High-quality reads were then aligned and quantified to the hg38 GENCODE reference database using STAR^74^ and an expectation/maximization (E/M) algorithm similar to the one in Xing *et al*^75^, respectively. Noncoding transcripts and protein-coding transcripts with an average read count < 10 per sample were removed; the remaining protein-coding transcript-level counts were summed to gene level and normalized with the median-of-ratios method used in DESeq2^76^. Expression profiles of each OTE group and 2D culture were compared to normal HBE profiles using human small airway epithelium transcriptome data previously published^44^. In brief, RNA-Seq data from five healthy nonsmokers was downloaded from the Sequence Read Archive (SRA Accession Number SRP005411) and processed using the same steps detailed above.

A pathway enrichment analysis of the significant protein-expressing transcripts (p < 0.05) identified in each OTE group and 2D culture was performed using NetworkAnalyst^50^. Lists were extracted and the resulting gene counts and p-value were included in tables.

### Statistical analysis

Data illustration and analysis were completed in GraphPad Prism 6 software and illustrated as mean ± SD. For each experiment, an n ≥ 3 was utilized for each experimental group. Statistical significance was determined with a minimum confidence interval of 95%. Specifically, a one-way ANOVA with Tukey’s multiple comparison post hoc test was used for all multiple comparison statistical analyses between the different hydrogel groups and 2D ALI culture. Meanwhile, an unpaired *t *test was performed to compare the difference in hydrogel stiffness with and without sECM. Histological and fluorescent images presented in figures were representative of their corresponding experimental groups.

## Supplementary Information

Supplementary Figures.

Supplementary Tables.

## Data Availability

The transcriptomic datasets generated for this study are available in the Gene Expression Omnibus (GSE207931).

## References

[1] Crystal RG, Randell SH, Engelhardt JF, Voynow J, Sunday ME (2008). Airway epithelial cells: Current concepts and challenges. Proc. Am. Thorac. Soc..

[2] Knight DA, Holgate ST (2003). The airway epithelium: Structural and functional properties in health and disease. Respirology.

[3] Hiemstra PS, McCray PB, Bals R (2015). The innate immune function of airway epithelial cells in inflammatory lung disease. Eur. Respir. J..

[4] De Rose V, Molloy K, Gohy S, Pilette C, Greene CM (2018). Airway epithelium dysfunction in cystic fibrosis and COPD. Mediat. Inflamm..

[5] Tam A, Wadsworth S, Dorscheid D, Man SP, Sin DD (2011). The airway epithelium: More than just a structural barrier. Ther. Adv. Respir. Dis..

[6] Sacco O (2004). Epithelial cells and fibroblasts: Structural repair and remodelling in the airways. Paediatr. Respir. Rev..

[7] Bagnato G, Harari S (2015). Cellular interactions in the pathogenesis of interstitial lung diseases. Eur. Respir. Rev..

[8] Kresse H, Schönherr E (2001). Proteoglycans of the extracellular matrix and growth control. J. Cell. Physiol..

[9] Liu L, Stephens B, Bergman M, May A, Chiang T (2021). Role of collagen in airway mechanics. Bioengineering.

[10] Cao X (2020). Invited review: Human air-liquid-interface organotypic airway tissue models derived from primary tracheobronchial epithelial cells—Overview and perspectives. Vitro Cell. Dev. Biol. Anim..

[11] Sakagami M (2020). In vitro, ex vivo and in vivo methods of lung absorption for inhaled drugs. Adv. Drug Deliv. Rev..

[12] Blume C, Davies DE (2013). In vitro and ex vivo models of human asthma. Eur. J. Pharm. Biopharm..

[13] Upadhyay S, Palmberg L (2018). Air-liquid interface: Relevant in vitro models for investigating air pollutant-induced pulmonary toxicity. Toxicol. Sci. Off. J. Soc. Toxicol..

[14] Hirst RA (2014). Culture of primary ciliary dyskinesia epithelial cells at air-liquid interface can alter ciliary phenotype but remains a robust and informative diagnostic aid. PLoS One.

[15] Fulcher ML, Gabriel S, Burns KA, Yankaskas JR, Randell SH (2005). Human Cell Culture Protocols.

[16] Jaroch K, Jaroch A, Bojko B (2018). Cell cultures in drug discovery and development: The need of reliable in vitro-in vivo extrapolation for pharmacodynamics and pharmacokinetics assessment. J. Pharm. Biomed. Anal..

[17] Barros AS, Costa A, Sarmento B (2020). Building three-dimensional lung models for studying pharmacokinetics of inhaled drugs. Adv. Drug Deliv. Rev..

[18] Myerburg MM (2007). Hepatocyte growth factor and other fibroblast secretions modulate the phenotype of human bronchial epithelial cells. Am. J. Physiol. Lung Cell. Mol. Physiol..

[19] Gaillard D, Puchelle E (1999). Lung Development.

[20] Nishioka M (2015). Fibroblast-epithelial cell interactions drive epithelial-mesenchymal transition differently in cells from normal and COPD patients. Respir. Res..

[21] Busch SM, Lorenzana Z, Ryan AL (2021). Implications for extracellular matrix interactions with human lung basal stem cells in lung development, disease, and airway modeling. Front. Pharmacol..

[22] Gu BH, Madison MC, Corry D, Kheradmand F (2018). Matrix remodeling in chronic lung diseases. Matrix Biol..

[23] Benam KH (2016). Small airway-on-a-chip enables analysis of human lung inflammation and drug responses in vitro. Nat. Methods.

[24] Benam KH (2016). Matched-comparative modeling of normal and diseased human airway responses using a microengineered breathing lung chip. Cell Syst..

[25] Sellgren KL, Butala EJ, Gilmour BP, Randell SH, Grego S (2014). A biomimetic multicellular model of the airways using primary human cells. Lab Chip.

[26] Plebani R (2021). Modeling pulmonary cystic fibrosis in a human lung airway-on-a-chip. J. Cyst. Fibros..

[27] Skardal A (2020). Drug compound screening in single and integrated multi-organoid body-on-a-chip systems. Biofabrication.

[28] Rajan SAP (2020). Probing prodrug metabolism and reciprocal toxicity with an integrated and humanized multi-tissue organ-on-a-chip platform. Acta Biomater..

[29] Sicard D (2018). Aging and anatomical variations in lung tissue stiffness. Am. J. Physiol. Lung Cell. Mol. Physiol..

[30] Eskandari M, Arvayo AL, Levenston ME (2018). Mechanical properties of the airway tree: Heterogeneous and anisotropic pseudoelastic and viscoelastic tissue responses. J. Appl. Physiol..

[31] Skardal A (2015). A hydrogel bioink toolkit for mimicking native tissue biochemical and mechanical properties in bioprinted tissue constructs. Acta Biomater..

[32] Albers S, Thiebes AL, Gessenich KL, Jockenhoevel S, Cornelissen CG (2015). Differentiation of respiratory epithelium in a 3-dimensional co-culture with fibroblasts embedded in fibrin gel. Multidiscipl. Respir. Med..

[33] Skardal A (2012). Tissue specific synthetic ECM hydrogels for 3-D in vitro maintenance of hepatocyte function. Biomaterials.

[34] Xu, D., Prestegard, J. H., Linhardt, R. J. & Esko, J. D. Proteins that bind sulfated glycosaminoglycans (2022). 35536933

[35] Munakata H, Takagaki K, Majima M, Endo M (1999). Interaction between collagens and glycosaminoglycans investigated using a surface plasmon resonance biosensor. Glycobiology.

[36] Tu Y, Mithieux SM, Annabi N, Boughton EA, Weiss AS (2010). Synthetic elastin hydrogels that are coblended with heparin display substantial swelling, increased porosity, and improved cell penetration. J. Biomed. Mater. Res. A.

[37] Kim M, Kim Y-J, Gwon K, Tae G (2012). Modulation of cell adhesion of heparin-based hydrogel by efficient physisorption of adhesive proteins. Macromol. Res..

[38] Pike DB (2006). Heparin-regulated release of growth factors in vitro and angiogenic response in vivo to implanted hyaluronan hydrogels containing VEGF and bFGF. Biomaterials.

[39] Coraux C, Roux J, Jolly T, Birembaut P (2008). Epithelial cell–extracellular matrix interactions and stem cells in airway epithelial regeneration. Proc. Am. Thorac. Soc..

[40] Natarajan V, Berglund EJ, Chen DX, Kidambi S (2015). Substrate stiffness regulates primary hepatocyte functions. RSC Adv..

[41] Polio SR (2019). Extracellular matrix stiffness regulates human airway smooth muscle contraction by altering the cell-cell coupling. Sci. Rep..

[42] Dominijanni AJ, Devarasetty M, Forsythe SD, Votanopoulos KI, Soker S (2021). Cell viability assays in three-dimensional hydrogels: A comparative study of accuracy. Tissue Eng. C Methods.

[43] Travaglini KJ (2020). A molecular cell atlas of the human lung from single-cell RNA sequencing. Nature.

[44] Hackett NR (2012). RNA-Seq quantification of the human small airway epithelium transcriptome. BMC Genom..

[45] Dvorak A, Tilley AE, Shaykhiev R, Wang R, Crystal RG (2011). Do airway epithelium air–liquid cultures represent the in vivo airway epithelium transcriptome?. Am. J. Respir. Cell Mol. Biol..

[46] Liu WK (2020). Protein profile of well-differentiated versus un-differentiated human bronchial/tracheal epithelial cells. Heliyon.

[47] Raidt J (2014). Ciliary beat pattern and frequency in genetic variants of primary ciliary dyskinesia. Eur. Respir. J..

[48] Lewis, M. & Stracker, T. H. Transcriptional regulation of multiciliated cell differentiation. *Semin. Cell Dev. Biol.* **110**, 51–60. 10.1016/j.semcdb.2020.04.007 (2021). 10.1016/j.semcdb.2020.04.007 32362381

[49] Ruiz García S (2019). Novel dynamics of human mucociliary differentiation revealed by single-cell RNA sequencing of nasal epithelial cultures. Development.

[50] Xia J, Benner MJ, Hancock RE (2014). NetworkAnalyst-integrative approaches for protein–protein interaction network analysis and visual exploration. Nucleic Acids Res..

[51] Mehra TD, Ghosh K, Shu XZ, Prestwich GD, Clark RA (2006). Molecular stenting with a crosslinked hyaluronan derivative inhibits collagen gel contraction. J. Investig. Dermatol..

[52] Ghosh K (2007). Cell adaptation to a physiologically relevant ECM mimic with different viscoelastic properties. Biomaterials.

[53] Gaetani R (2015). Epicardial application of cardiac progenitor cells in a 3D-printed gelatin/hyaluronic acid patch preserves cardiac function after myocardial infarction. Biomaterials.

[54] Varner VD, Gleghorn JP, Miller E, Radisky DC, Nelson CM (2015). Mechanically patterning the embryonic airway epithelium. Proc. Natl. Acad. Sci..

[55] Booth AJ (2012). Acellular normal and fibrotic human lung matrices as a culture system for in vitro investigation. Am. J. Respir. Crit. Care Med..

[56] Burgess JK, Mauad T, Tjin G, Karlsson JC, Westergren-Thorsson G (2016). The extracellular matrix–the under-recognized element in lung disease?. J. Pathol..

[57] Pageau SC, Sazonova OV, Wong JY, Soto AM, Sonnenschein C (2011). The effect of stromal components on the modulation of the phenotype of human bronchial epithelial cells in 3D culture. Biomaterials.

[58] Kreimendahl F (2019). Combination of vascularization and cilia formation for three-dimensional airway tissue engineering. J. Biomed. Mater. Res. A.

[59] Wolf MT (2012). A hydrogel derived from decellularized dermal extracellular matrix. Biomaterials.

[60] Mak KM, Mei R (2017). Basement membrane type IV collagen and laminin: An overview of their biology and value as fibrosis biomarkers of liver disease. Anat. Rec..

[61] Nowarski R, Jackson R, Flavell RA (2017). The stromal intervention: Regulation of immunity and inflammation at the epithelial-mesenchymal barrier. Cell.

[62] Goldstein AS (2013). A symbiotic relationship between epithelial and stromal stem cells. Proc. Natl. Acad. Sci..

[63] Antunes MB, Cohen NA (2007). Mucociliary clearance–a critical upper airway host defense mechanism and methods of assessment. Curr. Opin. Allergy Clin. Immunol..

[64] Warner SM (2013). Transcription factor p63 regulates key genes and wound repair in human airway epithelial basal cells. Am. J. Respir. Cell Mol. Biol..

[65] Zuo W-L (2018). Ontogeny and biology of human small airway epithelial club cells. Am. J. Respir. Crit. Care Med..

[66] Bukowy-Bieryłło Z (2021). Long-term differentiating primary human airway epithelial cell cultures: How far are we?. Cell Commun. Signal..

[67] Saint-Criq V (2020). Choice of differentiation media significantly impacts cell lineage and response to CFTR modulators in fully differentiated primary cultures of cystic fibrosis human airway epithelial cells. Cells.

[68] Vila A (2020). Hydrogel co-networks of gelatine methacrylate and poly (ethylene glycol) diacrylate sustain 3D functional in vitro models of intestinal mucosa. Biofabrication.

[69] Pereira C, Araújo F, Barrias CC, Granja PL, Sarmento B (2015). Dissecting stromal-epithelial interactions in a 3D in vitro cellularized intestinal model for permeability studies. Biomaterials.

[70] Skardal A (2017). Multi-tissue interactions in an integrated three-tissue organ-on-a-chip platform. Sci. Rep..

[71] Gentzsch M (2017). Pharmacological rescue of conditionally reprogrammed cystic fibrosis bronchial epithelial cells. Am. J. Respir. Cell Mol. Biol..

[72] Liu X (2012). ROCK inhibitor and feeder cells induce the conditional reprogramming of epithelial cells. Am. J. Pathol..

[73] Martin M (2011). Cutadapt removes adapter sequences from high-throughput sequencing reads. EMBnet. J..

[74] Magoč T, Salzberg SL (2011). FLASH: Fast length adjustment of short reads to improve genome assemblies. Bioinformatics.

[75] Jang JS (2020). Comparative evaluation for the globin gene depletion methods for mRNA sequencing using the whole blood-derived total RNAs. BMC Genom..

[76] Love MI, Anders S, Huber W (2017). Analyzing RNA-seq data with DESeq2. Bioconductor.

