# Supplementary material for: Development of a novel air–liquid interface airway tissue equivalent model for in vitro respiratory modeling studies
Source: Sci Rep. 2023 Jun 22;13:10137. doi: 10.1038/s41598-023-36863-1 (PMC10287689; doi:10.1038/s41598-023-36863-1)
Supplement: Supplementary file 1 — Supplementary Figures. [file 41598_2023_36863_MOESM1_ESM.pdf]

Development of a Novel Air-Liquid Interface Airway Tissue Equivalent Model for In Vitro Respiratory Modeling Studies

Timothy Leach[1,2], Uma Gandhi[1], Kimberly D. Reeves[3], Kristina Stumpf[1], Kenichi Okuda[4], Frank C. Marini[1], Stephen J. Walker[1], Richard Boucher[4], Jeannie Chan[3], Laura A. Cox[3], Anthony Atala[1,2], Sean V. Murphy[1,2]*

*Primary correspondence to:

Sean V. Murphy

391 Technology Way

Winston-Salem, NC 27101

semurphy@wakehealth.edu

1. Wake Forest Institute for Regenerative Medicine, Wake Forest School of Medicine, Medical Center, Winston-Salem, NC, 27101, USA
2. Virginia Tech-Wake Forest School of Biomedical Engineering and Sciences, Wake Forest School of Medicine, Medical Center Boulevard, Winston-Salem, NC, 27157, USA
3. Center for Precision Medicine, Department of Internal Medicine, Wake Forest School of Medicine, Winston-Salem, NC, 27157, USA
4. Marsico Lung Institute/Cystic Fibrosis Research Center, University of North Carolina at Chapel Hill, Chapel Hill, NC, 27599, USA

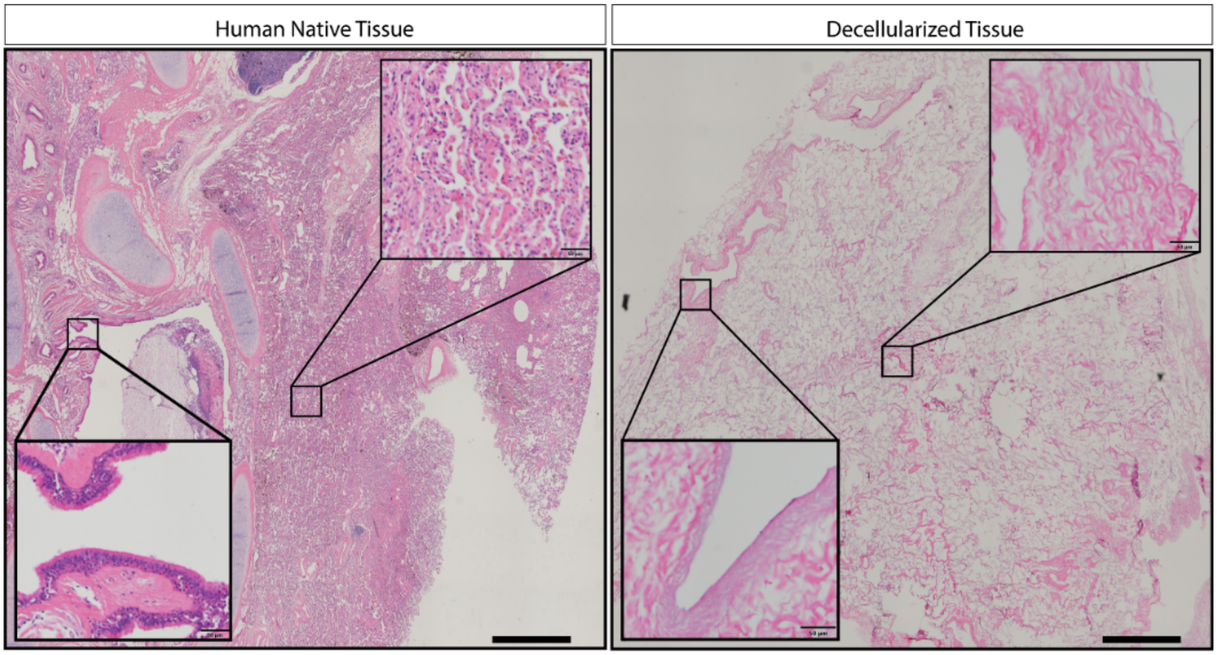

Supplementary Fig S1: H&E Imaging of Decellularized Lung Tissue. Comparison of native human lung tissue and decellularized human lung tissue demonstrating loss of cellular components in decellularized tissue, as shown by hematoxylin and eosin (H&E) staining.

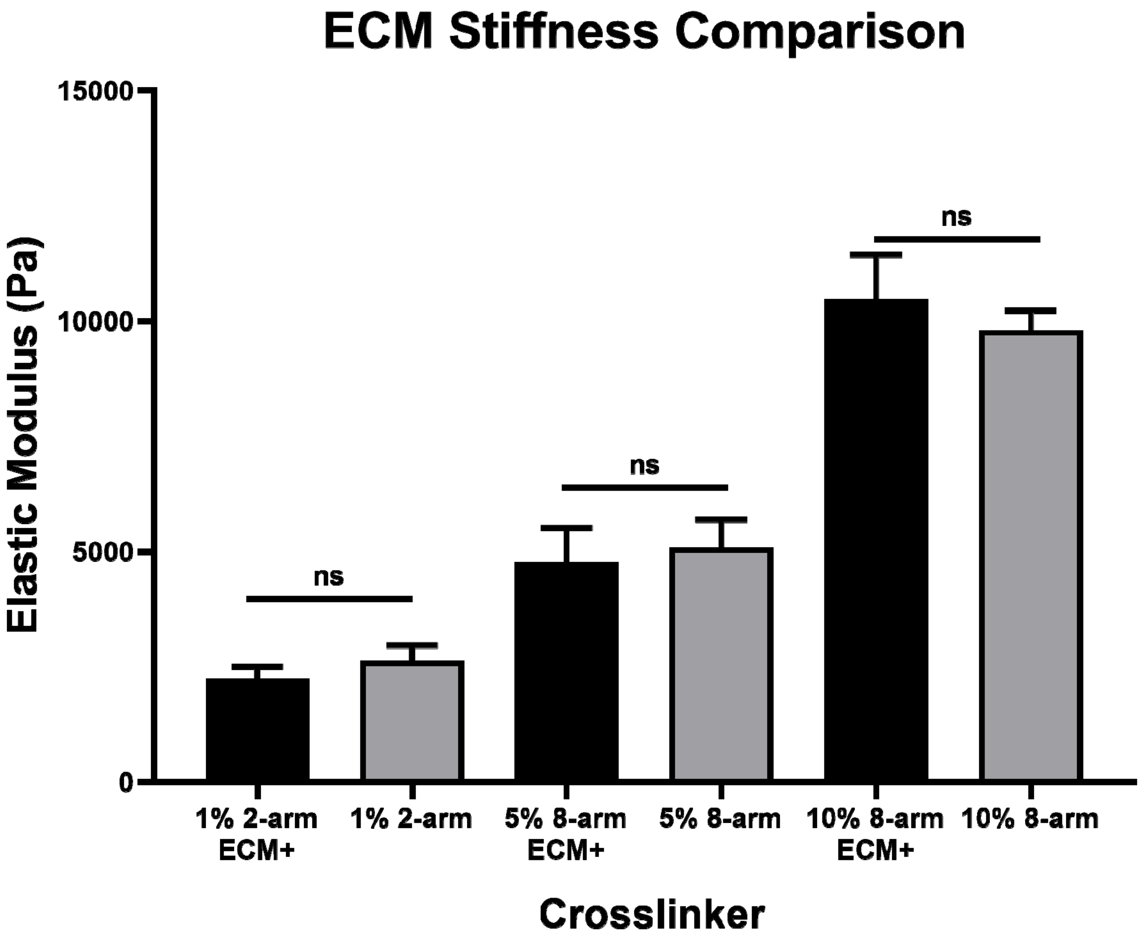

Supplementary Fig S2: sECM Hydrogel Stiffness Comparison. Elastic modulus quantification of the three crosslinked hydrogel groups with and without the sECM added into the hydrogel to demonstrate no significant difference in stiffness (n=6) (ns = not significant).

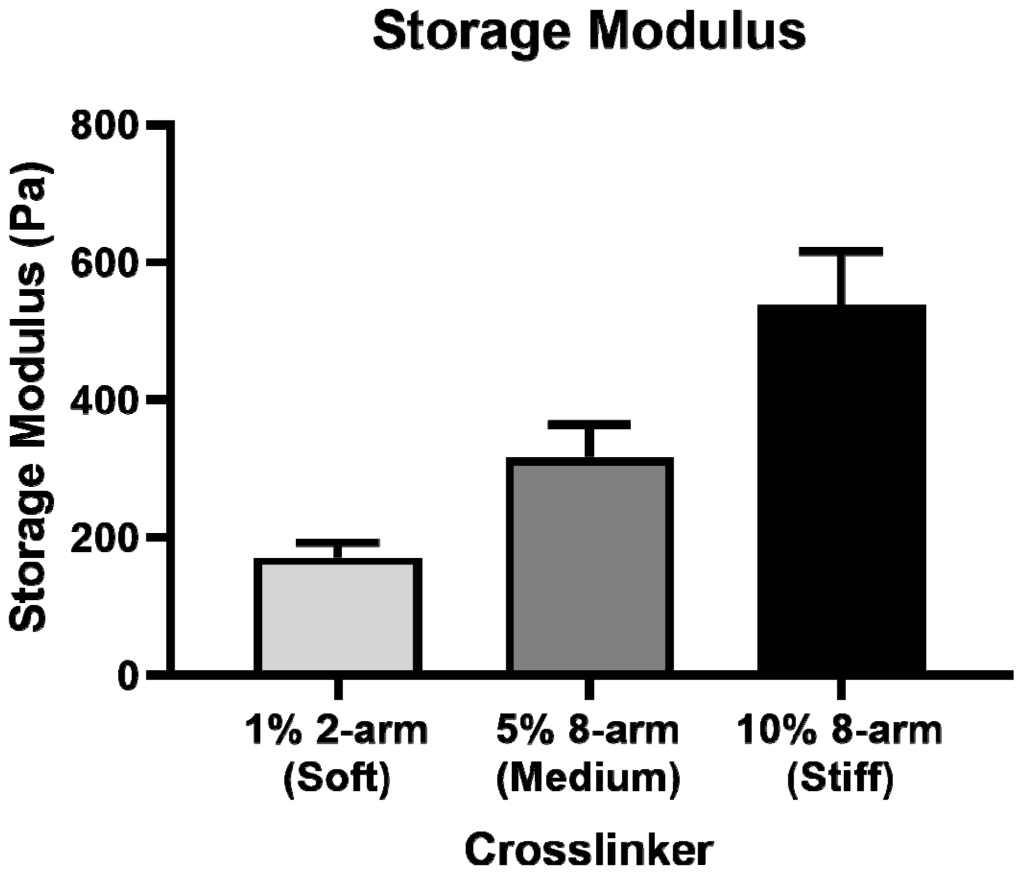

Supplementary Fig S3: Storage Modulus of Hydrogel. The storage modulus was quantified for each hydrogel stiffness group and displayed a similar trend of increasing storage modulus across the crosslinking concentrations (n=6).

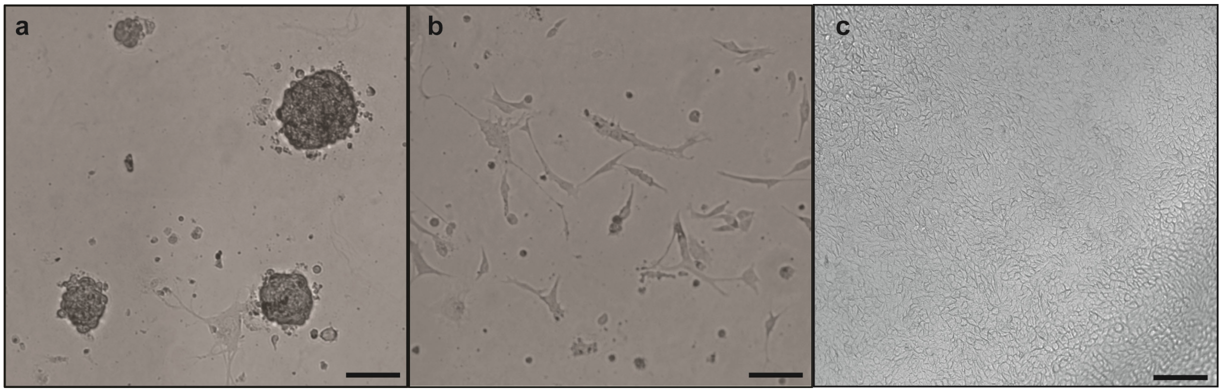

Supplementary Fig S4: Unhealthy vs Healthy Epithelial Whole Mount Imaging. Whole mount imaging of the unhealthy epithelial surface of the unsuccessful hydrogel groups showed HBE cells **(A)** attaching to each other in spherical shapes or **(B)** stretching out instead of the typical cobblestone-shaped phenotype. **(C)** Image of healthy confluent monolayer of HBE cells (Scale bar = 100 μm).

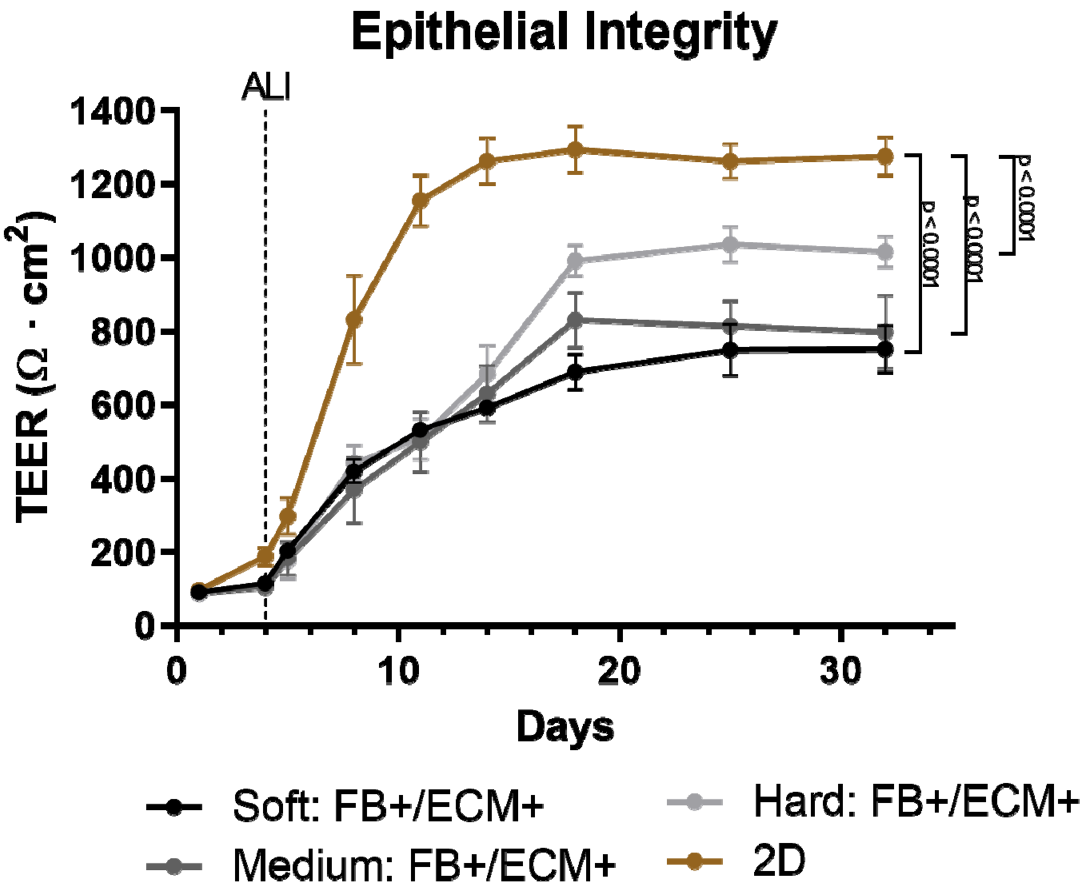

Supplementary Fig S5: Trans-Epithelial Electrical Resistance Comparison. Trans-epithelial electrical resistance (TEER) was additionally completed on 2D ALI cultures and compared against the 3D OTE models at the three tested stiffnesses. The final TEER value of the 2D ALI group was significantly higher than all three complete 3D OTE models (n=12).

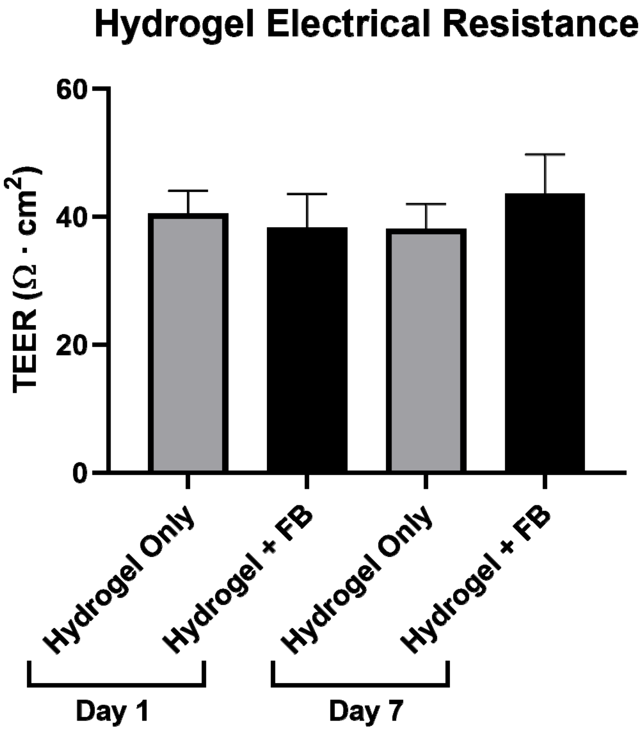

Supplementary Fig S6: Electrical Resistance of 3D OTE Hydrogel. Trans-epithelial electrical resistance was measured for hydrogel samples with and without fibroblasts to confirm they did not contribute to the TEER measurements of the 3D OTE samples. There was no significant difference between samples or between days.

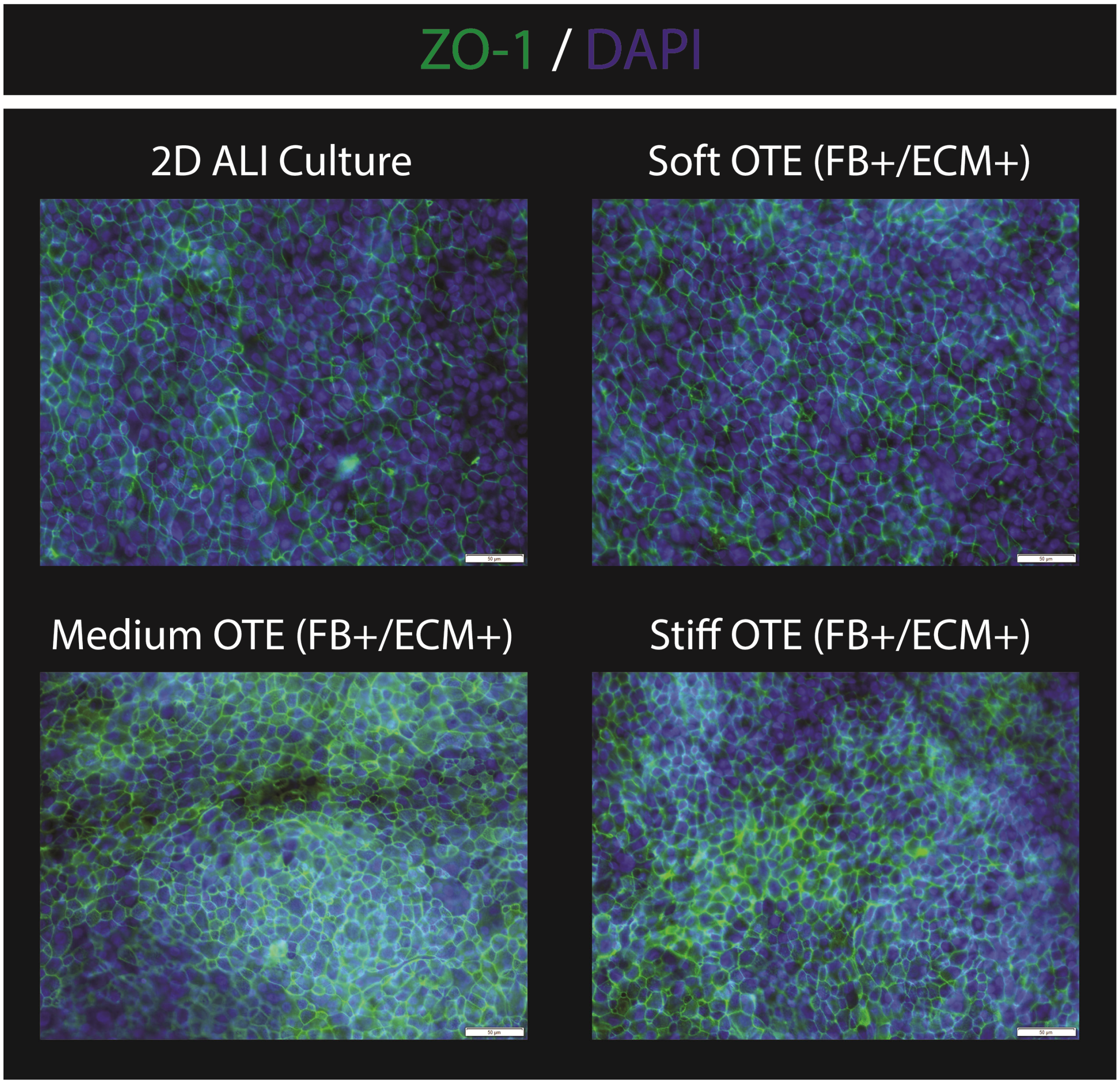

Supplementary Fig S7: Epithelial Tight Junction Staining. Whole mount immunostaining of tight junction marker ZO-1 (green) and DAPI (blue) of representative 3D OTE groups and 2D ALI culture.

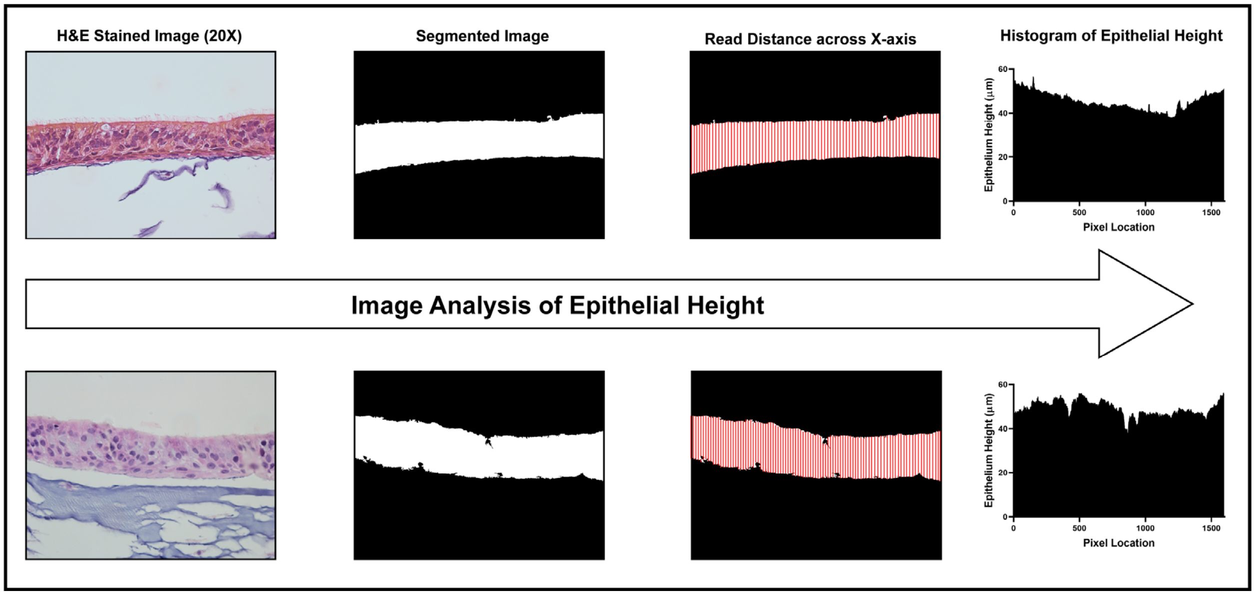

Supplementary Fig S8: MATLAB Analysis of Epithelial Height. To quantify the epithelial height of the 2D and 3D OTE cultures, H&E images were segmented in MATLAB to isolate the epithelium and the thickness of the area was measured across each x-axis pixel and averaged for a final measurement.

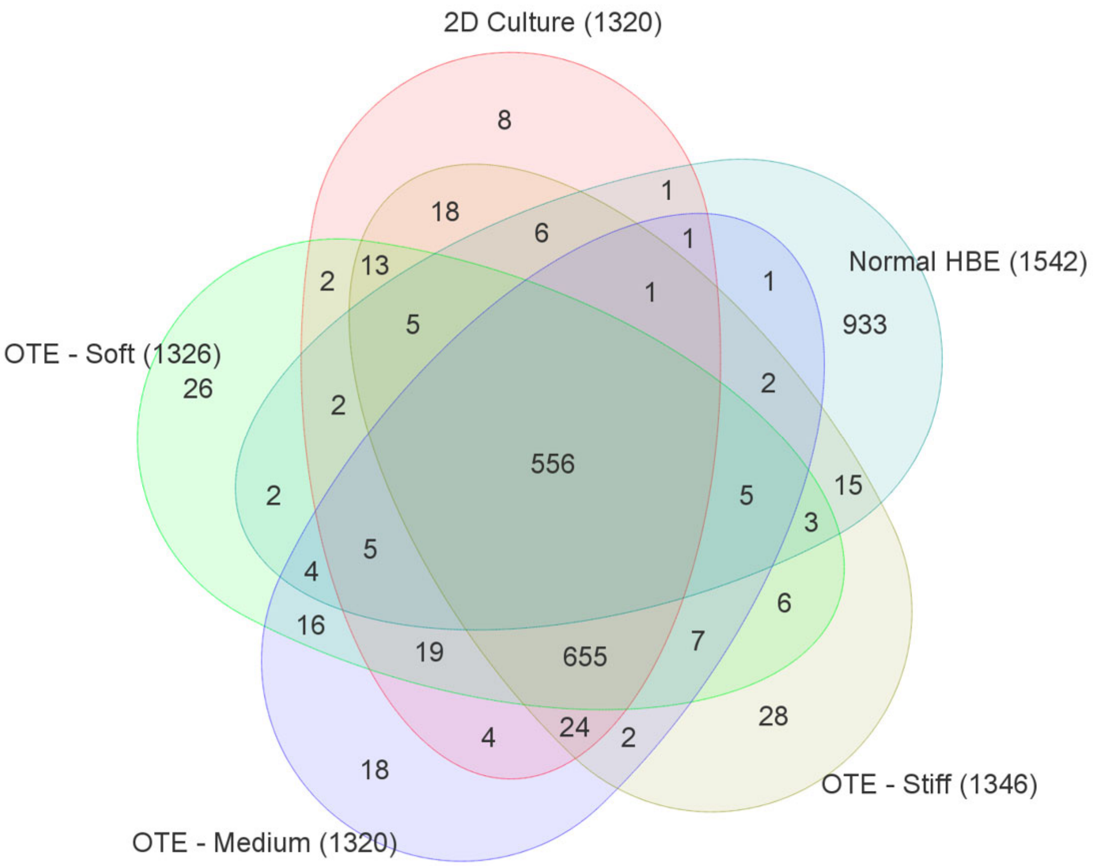

Supplementary Fig S9. *Complex Venn Diagram of Top Transcripts.* Venn diagram comparing the top 10% gene expression of the three stiffness 3D OTE models, 2D ALI culture, and *in vivo* human airway epithelium (Normal HBE).
